# Integrated data analysis allows the establishment of a new, cosmopolitan genus of marine Macrodasyida (Gastrotricha)

**DOI:** 10.1038/s41598-019-43977-y

**Published:** 2019-05-29

**Authors:** M. Antonio Todaro, Matteo Dal Zotto, Tobias Kånneby, Rick Hochberg

**Affiliations:** 1 Dipartimento di Scienze della Vita, Università di Modena and Reggio Emilia, Via Giuseppe Campi, 213/D, I-41125 Modena, Italy; 2 Consorzio per il Centro Interuniversitario di Biologia Marina e Ecologia Applicata ‘G Bacci’, v.le Nazario Sauro 4, I-57128 Livorno, Italy; 3 Lillhagsskolan, Depkens väg 21, 804 26 Gävle, Sweden; 4 University of Massachusetts Lowell, One University Avenue, Lowell, MA 01854 USA

**Keywords:** Zoology, Evolution

## Abstract

Macrodasyida (phylum Gastrotricha) comprises 365 species distributed across 34 genera and 10 families. However, current classification is under revision due to the contradictory results of molecular and morphological cladistic analyses. Studies aimed at bridging the gaps took advantage of supplementary assessments of poorly known species and particularly from observations of new taxa showing original traits that could help to identify plesiomorphic character states in these anatomically diverse micrometazoa. We follow this path by describing three new interesting macrodasyidan species respectively from Italy, Brazil and Sweden. In many respects, the new species resemble most closely species of the genus *Macrodasys*; however, details of the external morphology, in combination with the different lay-out of the reproductive system and the tiny spermatozoa lacking a visible flagellum, suggest they belong to a new genus, possibly in the family Macrodasyidae. These hypotheses are supported by the phylogenetic relationships of 47 taxa inferred from analyses of the 18S rRNA gene, which found the new species clustering with *Thaidasys tongiorgii* in a subset of a larger clade containing *Macrodasys*. Accordingly, the establishment of the following taxa is proposed: *Kryptodasys* gen. nov., *K*. *marcocurinii* sp. nov., *K*. *carlosrochai* sp. nov. and *K*. *ulfjondeliusi* sp. nov.

## Introduction

Gastrotrichs are microscopic, benthic invertebrates found in both freshwater and marine ecosystems worldwide^1^. They constitute a phylum, recently united with the Platyhelminthes in a clade named Rouphozoa^2,3^. Gastrotricha includes, as of February 2019, 852 species, 511 of which are marine and 341 freshwater^4^. Gastrotricha is divided into two orders: Chaetonotida, common in both freshwater and marine ecosystems, and Macrodasyida, which is mostly marine^5,6^.

Macrodasyida includes about 370 interstitial species living in littoral and/or sublittoral sandy habitats. Classification and α-biodiversity of the entire order are in a state of flux as shown by the recent ingroup taxa reassessments based on new phylogenetic data^7–9^ and the persistent description of new species^10–15^. Taxonomic novelties have also emerged at supraspecific levels; in fact over the last five-six years, the number of genera and families has increased to 35 and 10, respectively, from the 31 genera and 8 families known before 2012^8,9,16^. The two genera described most recently, *Hummondasys* Todaro, Leasi & Hochberg, 2014 and *Thaidasys* Todaro, Dal Zotto & Leasi, 2015, were established on the base of peculiar specimens found in geographic areas never investigated before with regard to the gastrotrich fauna. Both genera are monotypic and so far are only known from their respective type locality: *Hummondasys* found at Negril in Jamaica, and *Thaidasys* collected at Phuket island in Thailand^8,9^. Curiously, among Chaetonotida, two genera were also recently described from areas poorly known, and similarly to the macrodasyidans, these genera are monotypic or nearly so: *Bifidochaetus* Kolicka & Kisielewski, 2015 and *Cephalonotus* Garraffoni, Araújo, Guidi, Laurenço & Balsamo, 2017^17,18^.

In a taxonomic framework, this scenario may lead to the wrong conclusion that nowadays relevant innovation regarding the Gastrotricha may originate only from remote areas and consist of rare and species-poor taxa having a restricted geographic distribution. In contrast with this vision, here we propose the establishment of a new genus based on the discovery of specimens belonging to a group of new species found in different regions of the world, including areas considered well investigated with regard to the gastrotrich fauna. The establishment of a new genus is based on the concordance between both the morphological characteristics of the studied specimens and the results of phylogenetic analyses based on the nucleotide sequence of the 18S rRNA gene. Furthermore, we show that species belonging to the new established genus were found in the past but they were erroneously affiliated to the genus *Macrodasys*.

## Results

### Taxonomic account

Phylum Gastrotricha Metschnikoff, 1865

Order Macrodasyida Remane, 1925 [Rao & Clausen, 1970]

Family Macrodasyidae Remane, 1924

Genus *Kryptodasys* gen. nov.

urn:lsid:zoobank.org:act:24D72D24-185C-8BCF-853C0E5023E

### Diagnosis

Body vermiform, up to 1000 μm in total length (TL), and up to 96 μm in breadth, vaulted dorsally and flattened ventrally, with vacuolated cells along the body margins; epidermal glands generally few, small, scattered along the body. Cuticle naked, without spines and/or scales. Head ovoid or slightly bulbous, bearing pestle organs in a noticeable constriction. Trunk broadest in the mid-gut region, narrowing gently to the anus, then more quickly to the caudum; caudum unilobed, more often in the form of a short tail. Sensorial cilia distributed singly in dorsolateral and lateral columns along the body, sparingly around the head. Ventral locomotory ciliation rather sparse, arranged into two bands that run separately along most of the body but converging posterior to the anus into a single band. Anterior adhesive tubes (TbA), up to six per side, forming diagonal columns, which originate straight from the body surface and project forward; ventral adhesive tubes (TbV), absent; lateral adhesive tubes (TbL), absent or up to 2 along the pharyngeal region; ventrolateral adhesive tubes (TbVL), up to 26 per side, some along the pharyngeal region but most in the intestinal region; dorsal adhesive tubes (TbD), absent or up to 11 per side, most of which are present along the intestinal region; (TbDL), absent; posterior adhesive tubes (TbP), up to 10, along the the caudal margins. Paired accessory adhesive tubes, of two-three tubes per side, arising ventrolaterally from a common base anterior to the pharyngo-intestinal junction. Mouth terminal, of mid-size (up to 25 μm in breadth), leading to a shallow buccal cavity (8–15 μm in length); pharynx up to 293 μm long and up to 31 μm wide; pharyngeal pores well distant from the base, with dorsolateral openings. Pharyngo-intestinal junction (PhIJ) at U31–U43. Intestine straight, slightly wider at mid body; anal opening ventral at U89–U93. Hermaphrodite; testicles paired, elongate, beginning just anterior to the PhIJ; sperm ducts presumably open separately on the ventral surface; spermatozoa, stubby (5–10 μm in length), rod-like to elliptical in shape, apparently devoid of a flagellum. Ovary solitary, rather posterior in the trunk region; oocytes growing from posterior to anterior with largest element dorsal to the mid intestine. Caudal organ, bulky, posterior to the ovary; glando-muscular in nature and approximately bullet-shaped; it bears a canal with a single opening at the posterior end; frontal organ, inconspicuous, anterior to the largest oocyte; non muscular in nature and approximately round-shaped; usually containing some spermatozoa and secretory material. Type-species, *Kryptodasys marcocurinii* sp. nov.; other species, *K*. *carlosrochai* sp. nov., *K*. *celticus* (Hummon, 2008), *K*. *hexadactylis* (Rao, 1970), *K*. *nobskaensis* (Hummon, 2008), *K*. *remanei* (Boaden, 1963) and *K*. *ulfjondeliusi* sp. nov.

### Etymology

*Kryptodasys* (*kryptós* Gr = hidden and *dasýs* Gr, hairy) the first word alludes to the uniqueness of this taxon, which has been overlooked for a long time while the second is traditionally used in the name of most gastrotrich genera and refers to their thick ciliature.

*Kryptodasys marcocurinii* sp. nov.

urn:lsid:zoobank.org:act:26B5FC81-CEEB-4741-9BA9-99DD162232B3

(Figs 1–4)

**Figure 1:**
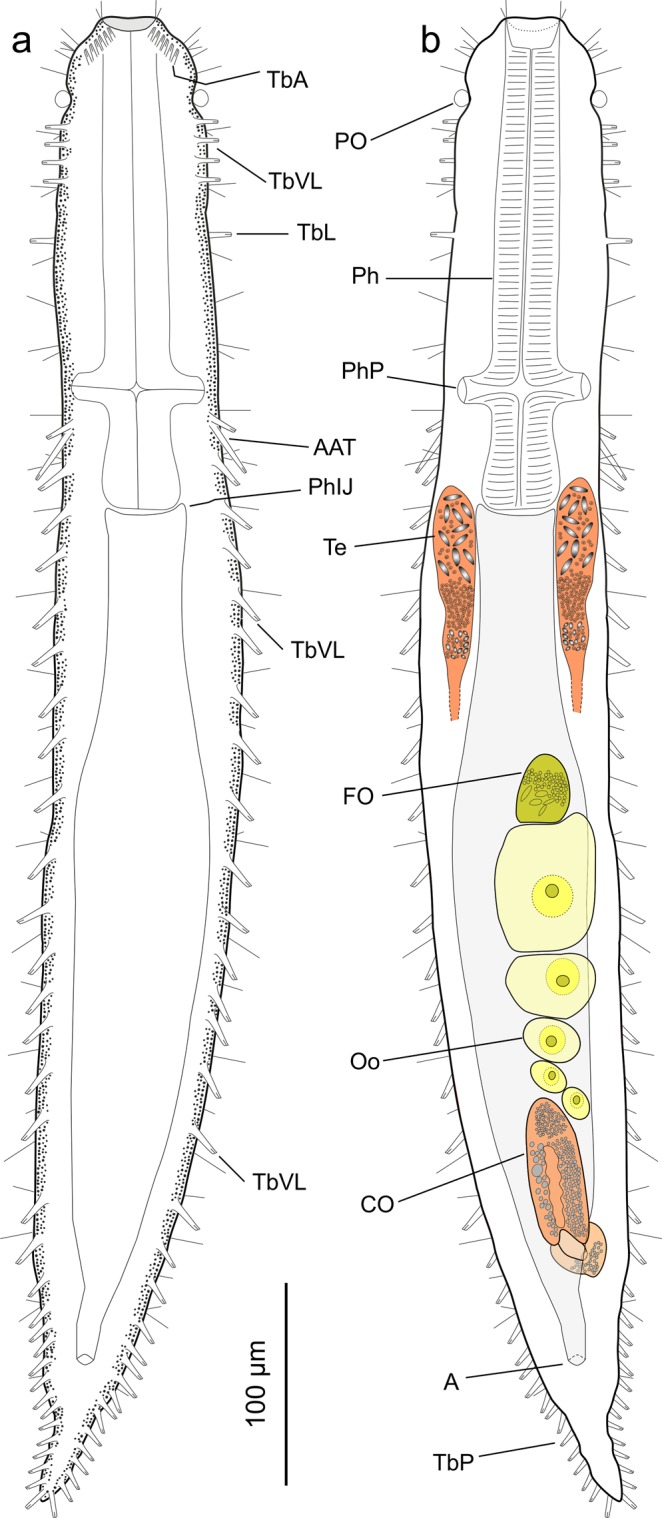
Line art illustrations of *Kryptodasys marcocurinii* sp. nov. (**a**) Habitus, ventral view. (**b**) Habitus, dorsal view, showing the internal organization with the male and female reproductive structures. Drawings are made mostly from the holotypic specimen. A = anus, AAT = accessory adhesive tubes, CO = caudal organ, FO = frontal organ, Oo = oocyte, Ph = pharynx, PhIJ = pharyngo–intestinal junction, Php = pharyngeal pore, PO = pestle organ, TbA = anterior adhesive tube, TbL = lateral adhesive tube, TbP = posterior adhesive tube, TbVL = ventrolateral adhesive tube, Te = testicle.

**Figure 2:**
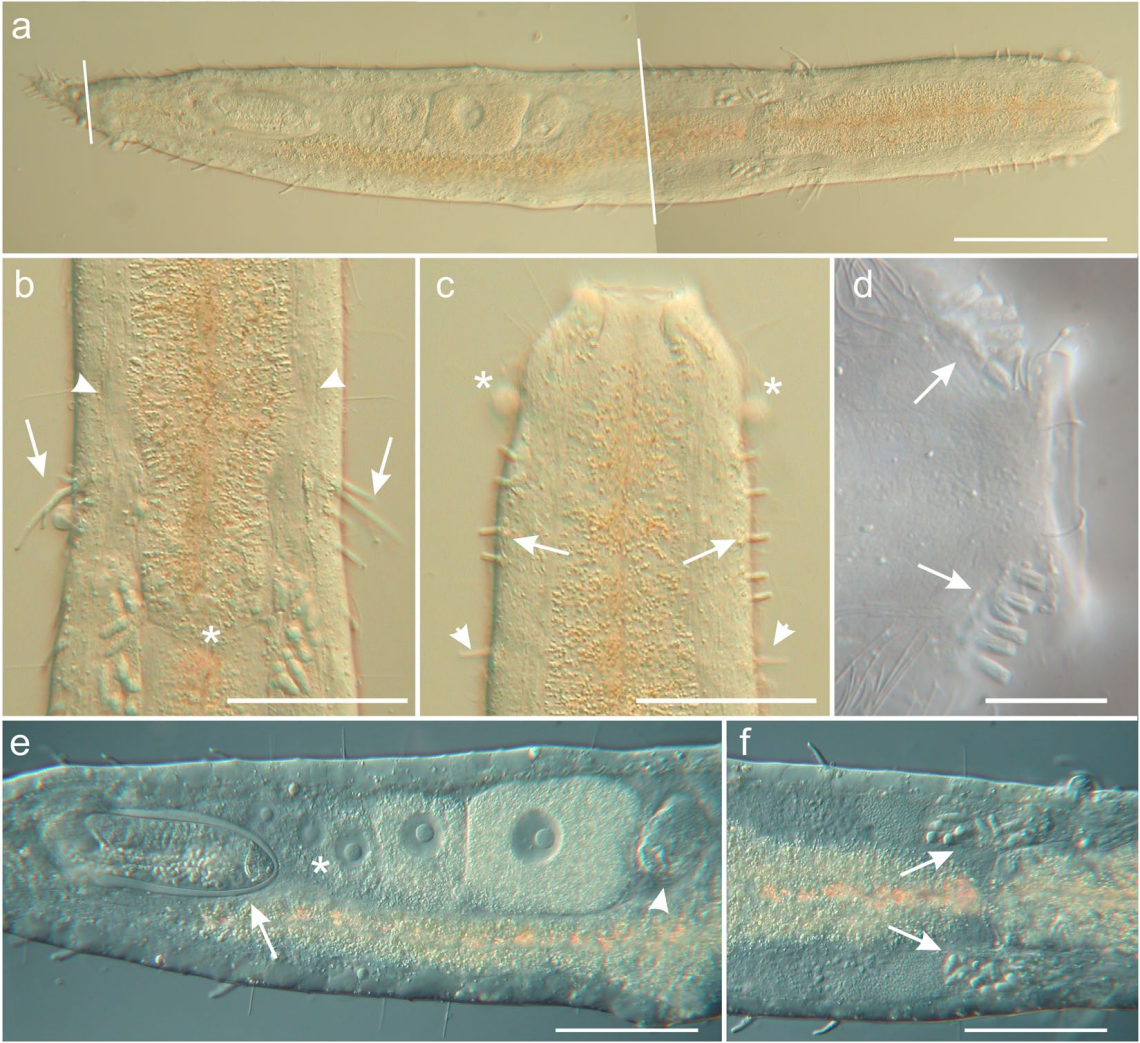
Differential interference contrast photomicrographs showing the morphology *of Kryptodasys marcocurinii* sp. nov. (**a**) Habitus, ventral view. (**b**) Close–up of the posterior pharynx region showing the accessory adhesive tubes (arrows), pharyngeal pores (arrowhead) and pharyngo-intestinal junction (asterisk). (**c**) Close-up of the anterior region, ventral view, showing the short ventrolateral adhesive tubes (arrows), lateral adhesive tubes (arrowhead) and pestle organs (asterisk). (**d**) Ventral view of the head, focussing on the anterior adhesive tubes (arrows). (**e**) Trunk region showing the ovary (asterisk), frontal organ (arrowhead) anterior to the largest egg, and caudal organ (arrows), posterior to the smallest oocyte. (**f**) Pharyngeo-intestinal body region, showing the testicles with spermatozoa (arrows). Scale bars (**a**) = 100 µm, (**b**,**c**,**e**,**f**) = 50 µm, (**d**) = 20 µm.

**Figure 3:**
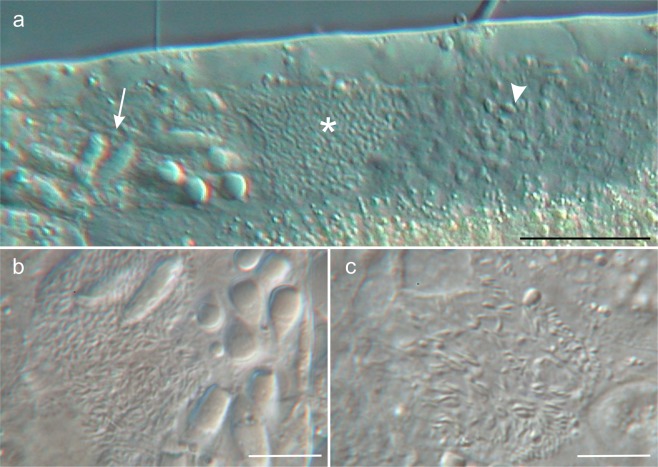
Differential interference contrast photomicrographs showing the male gonad of *Kryptodasys marcocurinii* sp. nov. (**a**) Left testicle apparently compartmentalized, showing mature spermatozoa (arrows) in the anterior portion, fine granular material (asterisks) in the mid portion, and small cellular elements, possibly maturing sperm, mixed with granular material in posterior region (arrowhead). (**b**) Close-up of the anterior portion. (**c**) Close-up of the posterior portion. Scale bars (**a**) = 50 µm, (**b**,**c**) = 10 µm.

**Figure 4:**
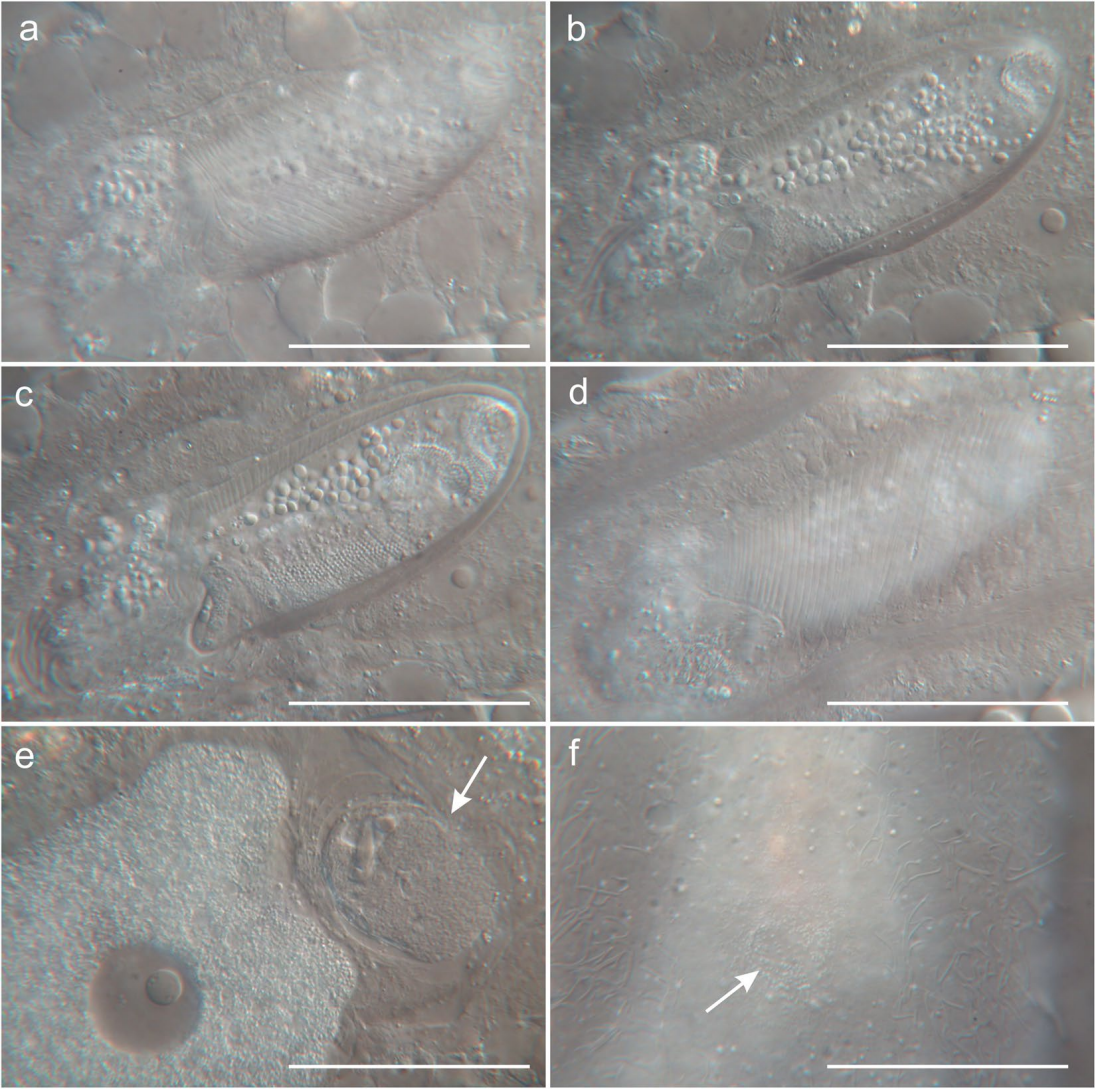
Differential interference contrast photomicrographs showing the accessory reproductive organs of *Kryptodasys marcocurinii* sp. nov. (**a**–**d**) Caudal organ at different focal planes. (**e**) Frontal organ, containing some spermatozoa and granular material. (**f**) External pore of the frontal organ on the ventral body side (arrow). Scale bars (**a**–**d**) = 50 µm, (**e**,**f**) = 30 µm.

### Diagnosis

Body elongate, 711–734 μm in total length (TL), and up to 96 μm in breadth; vaulted dorsally and flattened ventrally, with vacuolated cells along the body margins; epidermal glands generally few, small, scattered along the body. Cuticle naked, not forming spines and/or scales. Head slightly bulbous, bearing noticeable rounded pestle organs in a constriction. Trunk broadest in the mid-gut region, narrowing gently to the anus, then more quickly to the posterior end; caudum in the form of a short tail. Sensorial cilia organised singly in lateral and dorsolateral columns along the body, sparingly on the lateral sides of the head. Ventral locomotory ciliation rather sparse, arranged into two bands that run separately along most of the body but converging posterior to the anus into a single band. Anterior adhesive tubes (TbA), five to six per side, forming diagonal columns emerging directly from the body surface and projecting forward; ventral adhesive tubes (TbV) absent; ventrolateral adhesive tubes TbVL, up to 25 of which four to five are smaller and closer to each other in the anterior pharyngeal region (U07–U13), one in the posterior pharyngeal region (U31) and the other 19 equally spaced over the intestinal region from the pharyngo-intestinal junction (PhIJ) to the anus; lateral adhesive tubes (TbL), 1 per side in the pharyngeal region at U15; dorsolateral (TbDL) and dorsal adhesive tubes (TbD) absent; posterior adhesive tubes (TbP), 8–10, along the caudal margins. Paired accessory adhesive tubes (AAT), of two tubes per side, arising ventrolaterally from a common base anterior to the pharyngo-intestinal junction at U27. Mouth terminal, of mid-size (up to 26 μm in width), leading to a shallow buccal cavity (15 μm in length); pharynx up to 227 μm long and up to 36 μm wide; pharyngeal pores distant from the base with dorsolateral openings at U25. PhIJ at U33. Intestine straight, slightly wider at mid body; anal opening on the ventral side at U90. Hermaphrodite; testicles paired, elongate, beginning just anterior to the PhIJ; sperm ducts short, presumably open separately on the ventral surface; spermatozoa, stubby (9 × 3 μm), elliptical to pear-shaped, apparently devoid of a flagellum. Ovary solitary, rather posterior in the trunk region; oocytes growing from posterior to anterior with largest egg dorsal to the mid intestine at U59. Caudal organ, bulky, posterior to the ovary, centered at U78; glando-muscular in nature and approximately bullet-shaped (71 μm long × 26 μm wide); it bears a canal with a single opening at the posterior end; frontal organ, sac-like, anterior to the largest oocyte, centered at U52; non muscular in nature and approximately ovoid in shape (36 × 26 μm); usually containing some spermatozoa and secretory material.

### Etymology

The species is named after Marco Curini-Galletti, a colleague, friend, and superb scuba diver, who in the numerous, joined sampling trips, scornful of danger, has on several occasions threatened his life to collect our samples, including the present ones.

### Type material

Holotype: the 734 μm long adult specimen shown in Figs 2–4 no longer extant (International Code of Zoological Nomenclature^19^, Articles 73.1.1 and 73.1.4; see also recommendation 73G–J of Declaration 45 - Addition of Recommendations to Article 73^20^), collected on 11/07/2005. *Additional examined material*. three adults, one subadult and one juvenile specimen, collected from the type locality and other locations; all were observed alive and went destroyed during the observation. Two additional adults were preserved in a 95% ethanol solution and subsequently used for DNA analysis (see below and Supplementary Table S1).

### Distribution and ecology

Type locality - Sardinia: Grotta di Nereo (Nereo’s cave, Lat. 40°33′70.5′N, Long. 08°09′62.9″E); occasional in frequency of occurrence and numerous in abundance in medium, moderately sorted sand collected at a depth of 30.7 m. Other locations: Grotta il Porticato, occasional in frequency and scarce in abundance at 20 m depth in coarse moderately sorted sand; Costa Paradiso, sparse in frequency and rare in abundance at 35 m depth in very coarse, well sorted sand. In all cases, values of temperature and salinity of the pore water at the time of samplings were 13 °C and 38 PSU, respectively. Values of the granulometric parameters are reported in Supplementary Table S2.

### Description

Based mostly on the adult specimen with a total body length of 734 μm shown in Fig. 2. Body vermiform and of medium width (Fig. 2a); vaulted dorsally and flattened ventrally, bearing vacuolated cells along the lateral and dorsolateral body margins; epidermal glands few, small, scattered along the body. Cuticle smooth, not forming spines and/or scales. Head slightly bulbous, bearing noticeable pestle organs in a constriction at U05 (Figs 1 and 2a). Body of similar width in the anterior third increasing slightly in breadth to mid trunk and thereafter narrowing gently to the anus, and then more quickly to the caudum; caudum in the form of a short tail (Figs 1 and 2a). Widths of head/mid pharynx/PhIJ/trunk/anus/base of tail, and locations along the length of the body are as follows: 64/79/85/96/43/25 μm at U04/U16/U33/U55/U90/U93, respectively.

Ciliation: Sparse sensorial cilia (10–18 μm long) insert on the dorsal and ventrolateral margin of the head, in addition to about 25–30 other elements (15–22 μm long) organised singly in dorsolateral and lateral columns along the body. Ventral locomotor ciliature forms two longitudinal bands extending separately from under the head to the posterior trunk region but converging behind the anal opening into a single band; ciliary bands appear wider, denser and closer to each other along the pharyngeal region (Fig. 2b).

Adhesive tubes: TbA, six per side (6–8 μm in length), forming diagonal columns, emerging directly from the body surface and projecting anteriorly (Figs 1a and 2d); TbV, absent; TbVL, 25 per side, four–five of which are smaller (10–12 μm in length) and closer to each other in the anterior pharyngeal region from U07 to U13 (Figs 1a and 2b), one is in the posterior pharyngeal region at U31 and the remaining 19 (13–18 μm in length) equally spaced along the intestinal region, from the PhIJ to the anus; TbL, one per side (13 μm in length) in the pharyngeal region at U15 (Fig. 1); TbD and TbDL absent; TbP, ten per side (8–13 μm in length), surrounding the caudum (Figs 1a and 2a). In addition, there are paired accessory adhesive tubes, two tubes per side, arising ventrolaterally from a common base anterior to the pharyngo–intestinal junction at U27; tubes are of different size, and posterolaterally directed; the shortest tube, 13 μm in length, arises anteriorly while the longest is posterior and twice as long, 26 μm (Figs 1 and 2a,b).

Digestive tract: Mouth is terminal, 26 μm in width, with the mouth rim slightly protruding forward; buccal cavity rather shallow (15 μm in length) and lined with a thin cuticle (Figs 1 and 2a); pharynx, 227 μm in length, widens to 37 μm toward the rear up; pharyngeal pores open dorsolaterally distant from the base at U25. Pharyngo-intestinal junction (PhIJ) at U33. Intestine is straight and increases in breadth from the PhIJ to mid-body where reaches 66 μm in width and then gradually narrows toward the posterior body end; anal opening ventral at U90. In all of the examined specimens, the intestine contained yellowish/orange coloured material, probably biodetritus, but not diatom frustules (Fig. 2e,f).

Reproductive tract: Hermaphroditic; testicles paired and elongate; they begin just anterior to the PhIJ and span posteriorly for about 95 μm, from U31 to U46 (Figs 1b and 2a,f). Testicles appear to be anatomically and functionally compartmentalized as the anterior portion contains large and better structured cellular elements (likely spermatozoa), the mid portion shows fine granular material, while the posterior portion holds granular material mixed with small cellular elements (likely maturing sperm) (Fig. 3). Each testicle apparently seems to continue posteriorly with a short sperm duct, which presumably opens on the ventral surface. Ultrastructural studies are needed to ascertain the exact organization and function of testicles and ducts. Spermatozoa are stubby (9 μm in length and 3 μm in width), elliptical to pear-shaped, apparently lacking a flagellum (Figs 1b, 2f and 3a,b). Ovary single, rather posterior in the trunk region; oocytes growing from posterior to anterior with largest oocyte dorsal to the mid intestine at U59. (Figs 1b and 2a,e). Caudal organ noticeable, posterior to the ovary, centered at U78 (Figs 1b and 2a,e); glando-muscular in nature and approximately bullet-shaped, 85 long and 26 μm wide (Fig. 4a–d); it bears a canal with a single opening at its posterior end; the entire organ opens on the ventral surface, anterior to the anus at U83. Frontal organ, sac-like, anterior to the largest oocyte, centered at U52; non muscular in nature and roughly ovoidal in shape (26 μm long and 34 μm wide); usually containing some spermatozoa and secretory material (Figs 1b, 2a,e and 4e). An inconspicuous opening, surrounded by secretory material, was observed on the ventral surface in correspondence of the frontal organ; it is interpreted as its external pore (Fig. 4f); the internal pore of the frontal organ was not seen.

### Variability and remarks

The three additional measured adult specimens ranged from 711 to 720 μm in total length. All of them had fully developed male and female gonads, and accessory reproductive organs, similar to the holotype. The adhesive apparatus was also similar to that of the holotype, however, the 720 μm long adult had five TbA and only four TbVL in the anterior region of the pharynx. The observed subadult, 680 μm in total length, showed on each side four TbA, six TbP and 18 TbVL, four of which were in the anterior region and one in the posterior region of the pharynx; the lateral adhesive tubes present in the pharyngeal region of the adult were also present in the subadult; likewise present were the paired accessory adhesive tubes. By contrast, the subadult specimens was lacking gonads and frontal organ while the caudal organ was starting to become visible. The juvenile, 434 μm in total length, showed on each side three TbA, three TbP and six TbVL, one of which was present in the anterior pharyngeal region. The TbL and the accessory adhesive tubes were present also. Ontogenetic variations with regard to metric and meristic traits (e.g., number of adhesive tubes) are a normal occurrence during gastrotrich growth. However, our data also indicates variability among adults, although it is minimal; variation was observed in the total length and the number of TbA and TbVL present in anterior region of the pharynx. On the other hand, other traits appear to be present throughout all of the life stages (at least the documented ones), as is the case of the single TbL in the pharyngeal region and the paired accessory adhesive tubes, both present from the juvenile age to adulthood. This new species will be differentiated later in light of the other two new species descriptions (see Supplementary Material, Appendix S1).

### Phylogenetic analysis

**Figure 5:**
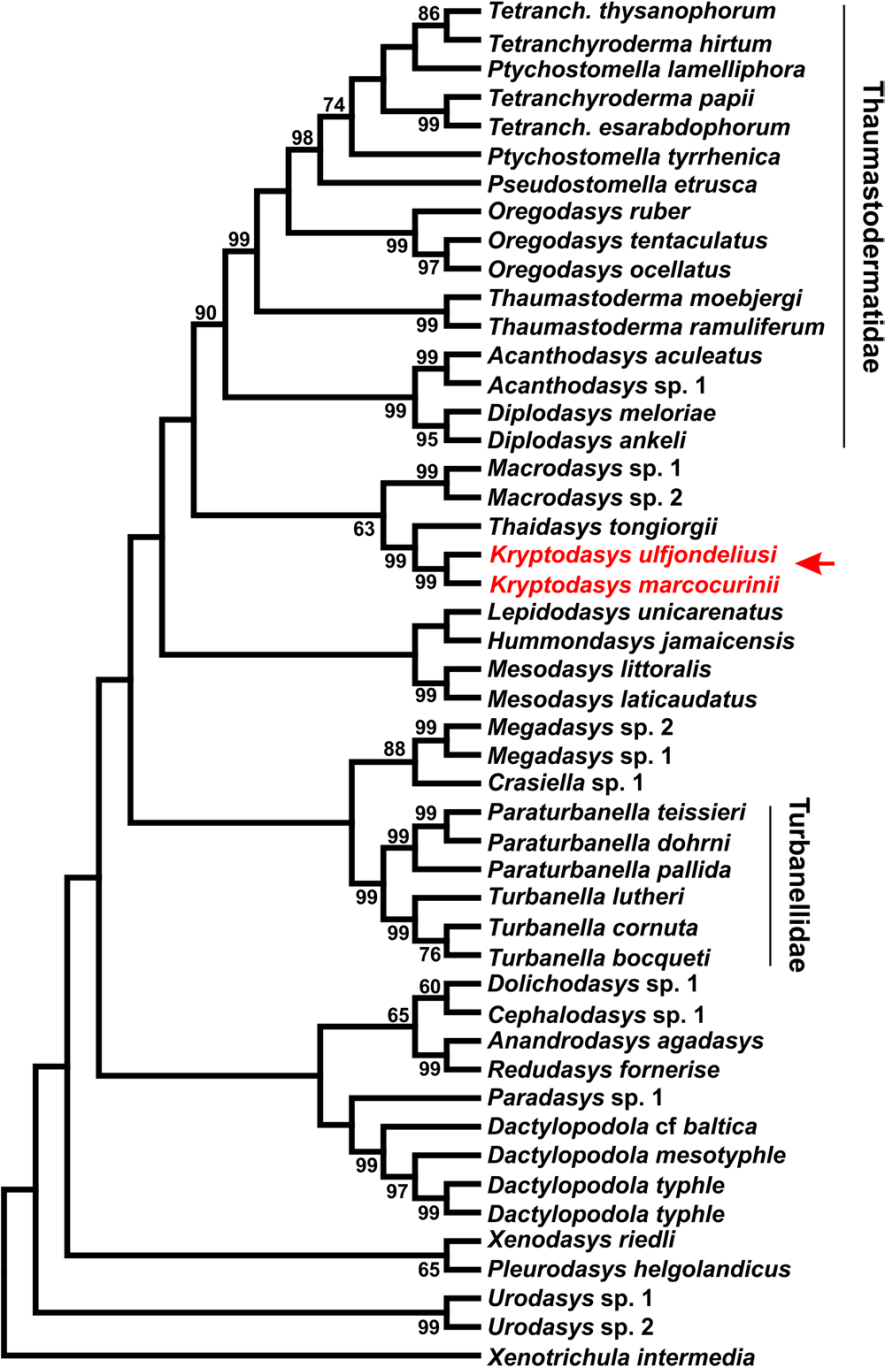
Phylogenetic relationships of 47 Gastrotricha Macrodasyida including two species of the new genus *Kryptodasys* inferred from MP analysis of 18S rRNA gene. The chaetonotidan *Xenotrichula intermedia* (Fam. Xenotrichulidae) is the outgroup. The most parsimonious tree (length = 4095; consistency index = 0.356007, composite index = 0.232281) is presented. Numerical values at nodes represent bootstrap support.

**Figure 6:**
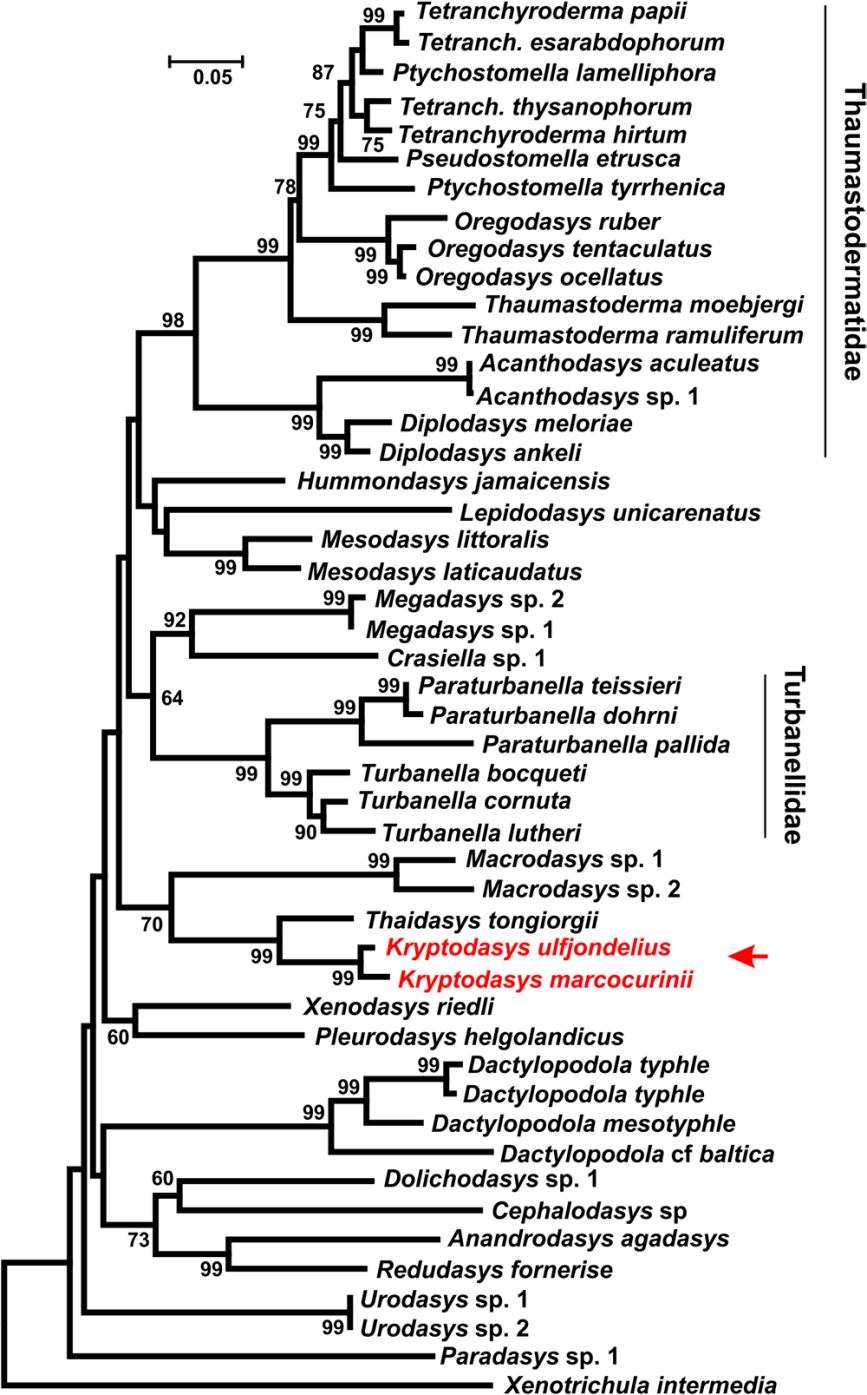
Phylogenetic relationships of 47 Gastrotricha Macrodasyida including two species of the new genus *Kryptodasys* inferred from ML analysis of 18S rRNA gene. The chaetonotidan *Xenotrichula intermedia* (Fam. Xenotrichulidae) is the outgroup. The tree with the highest log likelihood (−20472.3991) is presented. Numerical values at nodes represent bootstrap support.

**Figure 7:**
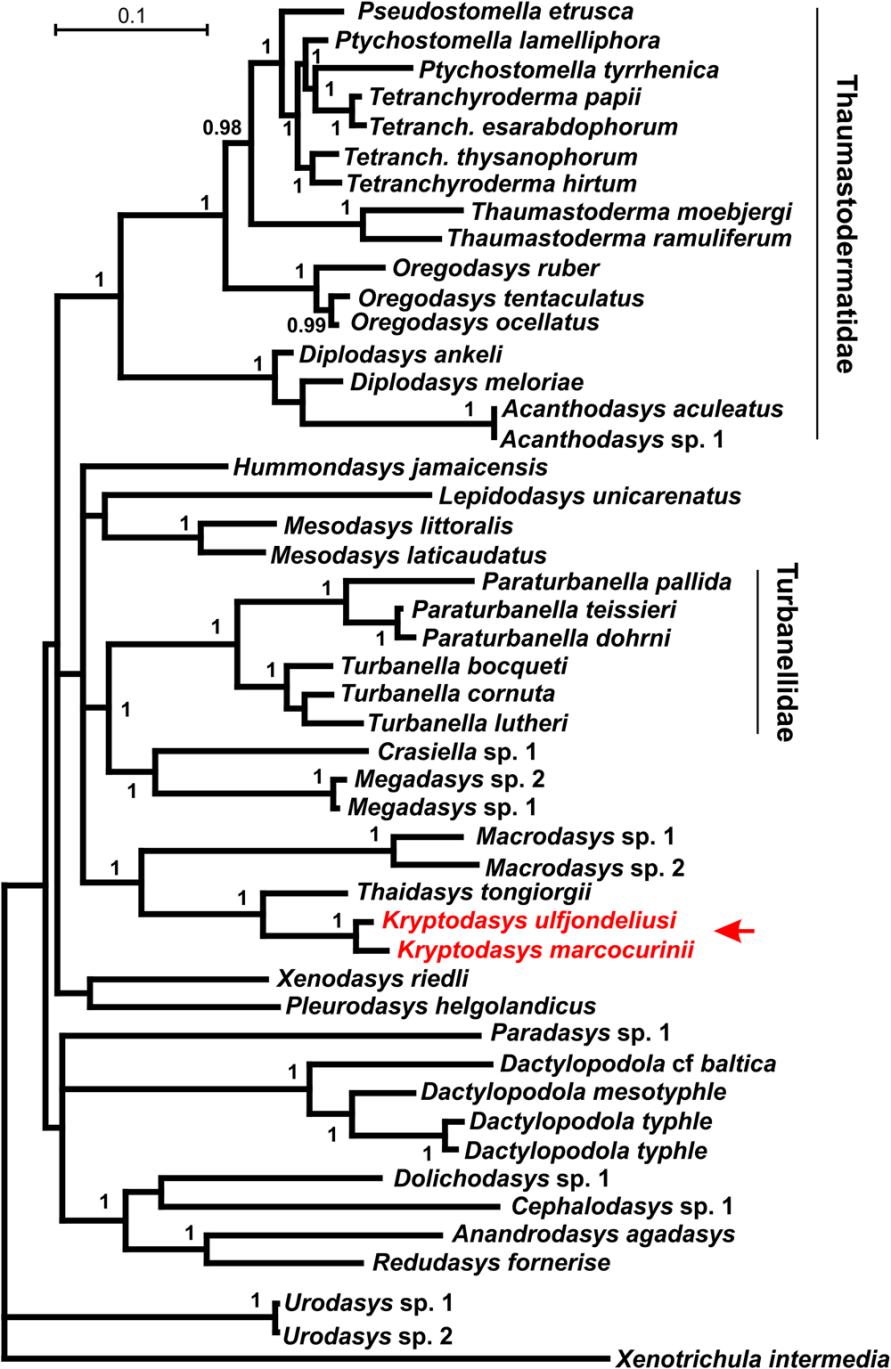
Phylogenetic relationships of 47 Gastrotricha Macrodasyida including two species of the new genus *Kryptodasys* inferred from Bayesian analysis of 18S rRNA gene. The chaetonotidan *Xenotrichula intermedia* (Fam. Xenotrichulidae) is the outgroup. Numerical values at nodes represent posterior probabilities.

The ultimate alignment counted 1906 positions, 850 of which appear constant and 723 informative under the parsimony criterion. MP, ML and Bayesian analysis yielded highly congruent topologies, with most of the shared clades bearing high statistic support at nodes: i.e., Bayesian posterior probability and bootstrap values >98% and >60, respectively (Figs 5–7).

Some of the well supported groups are (1) the heavily sampled families Turbanellidae and Thaumastodermatidae and their traditional subgroupings (i.e., subfamilies and genera); (2) the sister-group relationship between *Anandrodasys agadasys* (Hochberg, 2003) and *Redudasys fornerise* Kisielewski, 1987; and (3) the recently recognised alliance between *Megadasys* Schmidt, 1974 and *Crasiella* Clausen, 1968.

On the other hand, Cephalodasyidae and Macrodasyidae under no circumstances appear as monophyletic due to the association between representatives of different families and/or the scattering along the phylogenetic trees of their respective species. Multisampled genera appear also as monophyletic in the obtained topologies (except *Ptychostomella* Remane, 1926 and *Tetranchyroderma* Remane, 1926).

Concerning the two new taxa included in the genus *Kryptodasys* gen. nov., all of the obtained trees show them forming a monophyletic clade that is sister to *Thaidasys tongiorgii* (Fam. Macrodasyidae). In turn, the clade *Thaidasys* + *Kryptodasys* gen. nov. appears in a sister-group relationship with *Macrodasys* (Fam. Macrodasyidae); all these clades received high statistical support at nodes (Figs 5–7).

## Discussion

### Remarks on diagnostic features and morphology

Body size and shape, general organization of the adhesive apparatus, and the arrangement and composition of the reproductive apparatus of the three new taxa are comparable to species of the genus *Macrodasys*. However, the new species appear to be distinct from the overwhelming majority of *Macrodasys* species due to peculiarities of their reproductive apparati and the morphology of their spermatozoa. Specifically, the new species have the frontal organ (=seminal receptacle, female in function) located anterior to the largest egg (Figs 2e and 4e; Supplementary Figs S3c, S6b and S7c), while species of *Macrodasys* have the frontal organ located posterior to the largest egg. Anatomically speaking, this means that while the frontal organ and caudal organ (=copulatory organ, male in function) of the new taxa are separated by the ovary and its cellular products (maturing oocytes), in *Macrodasys* the two organs are positioned next to each other in the same body compartment. Regarding the male gametes, the new species possess rather short, smooth spermatozoa lacking a regular flagellum (Figs 2f and 3a,b; Supplementary Figs S3a,c, S6b and S7b), in contrast with the species of *Macrodasys*, which possess filiform sperm, with a cork-screw-like anterior portion and a long flagellum showing the usual 9 × 2 + 2 axonemal organization^21^.

Studies have shown that the highly differentiated shape of the accessory reproductive organs, the organization of the reproductive apparatus, and the structure and ultrastructure of the spermatozoa bear phylogenetic signals that may be utilized, usefully, for taxonomic and classification purposes^7,22,23^. Thus, in our opinion, the morphological differences highlighted above are indicative of a clear distinctness (autoapomorphies) of the new taxa from *Macrodasys* and suggest the establishment of a new genus to accommodate them. The phylogenetic analyses based on a molecular marker (18S rRNA gene) provides support for such a hypothesis (see phylogenetic results above and phylogenetic remarks below). Therefore, the establishment of the new taxon, *Kryptodasys* gen. nov. is proposed.

The three new described species (see also Supplementary Material, Appendix S1), can easily be distinguished from each other based on several autoapomorphic characteristics of the adult specimens. Some of the main distinctive traits are reported hereafter (see also Supplementary Table S3). *K*. *marcocurinii* sp. nov. is of larger size, compared to *K*. *carlosrochai* sp. nov. and to *K*. *ulfjondeliusi* sp. nov.; for instance, the Sardinian species has a TL up to 734 µm while the other two species are 515 µm and 595 µm in total length, respectively. The Sardinian species also has many more adhesive tubes compared to the Brazilian and the Swedish species; for example, in the former species, the TbA are up to 6 per side vs 3 and 4 tubes present, respectively, in the other two species; there are up to 25 TbVL in the Sardinian species vs 10 and 18 tubes present in the Brazilian and Swedish taxa, respectively. The arrangement of the TbVL is also different in the three species. More specifically, *K*. *marcocurinii* shows 5–6 tubes in the pharyngeal region, 4–5 of which are of smaller size and closer to each other in the anterior region; in *K*. *carlosrochai* there is a single tube in the pharyngeal region located halfway along the pharynx and its size is not different from the other TbL; in *K*. *ulfjondeliusi* there are three tubes in the pharyngeal region of ‘normal’ size that are equally spaced along the posterior half of the pharynx. Moreover, *K*. *carlosrochai* is unique also in that its accessory adhesive tubes are made up of 3 tubes each while in the other two species these organs are made up of 2 tubes only. *K*. *ulfjondeliusi* is further distinguishable as it possesses TbD, which are absent in the other two species.

### A revision of *Macrodasys* species

Currently, the genus *Macrodasys* includes 37 species, making it the second largest macrodasyidan genus after *Tetranchyroderma* (Thaumastodermatidae). *Macrodasys* was established by Adolf Remane based on the distinctive morphological characteristics of a novel ‘….aberrante Gastrotrichen…’ found in the bay of Kiel (Germany), named *Macrodasys buddenbrocki*^24^. *M*. *buddenbrocki* Remane, 1924 was described and illustrated as having paired testicles filled with filiform spermatozoa, and two accessory reproductive organs, the most anterior of which is located posterior to the largest egg cells (see Pg. 24, Fig. 2 in Remane^24^). This organ, described as being connected to the sperm ducts, was thought by Remane to function as a *vesicula seminalis*, thus being male in function. It is now known that in *Macrodasys* sperm ducts empty separately on the ventrolateral sides via independent pores; consequently, Remane’s *vesicula seminalis* is currently interpreted as a seminal receptacle, thus female in function, and it is called a frontal organ^25^. The second accessory sexual organ of *M*. *buddenbrocki* is located more posterior in the trunk region. This additional organ, considered by Remane to be a *bursa copulatrix* (female in function), is currently interpreted as a copulatory organ and is called a caudal organ^25^.

Most of the species presently affiliated to the genus *Macrodasys* have been described as having such traits, including the four additional species described by Remane himself in the years following the discovery of *M*. *buddenbrocki*^26–28^. However, some *Macrodasys* species, described later on by other authors, do not appear to comply fully with the organization of the reproductive system typical of the genus. These species include: *M*. *remanei* Boaden, 1963 from north Wales (U.K.), *M*. *hexadactylis* Rao, 1970 from the coast of Andhra (India) and *M*. *nobskaensis* Hummon, 2008 from Massachusetts (USA). One of the biggest differences is that in these three species, a structure interpretable as the frontal organ is positioned in front to the largest egg cells as opposed to posterior to the egg cells characteristic of other species of *Macrodasys*^29–31^. In our opinion, this evident discrepancy distinguishes these three species from the ‘genuine’ species of the genus *Macrodasys;* the general morphology of these species is also more similar to our new species. Consequently, we propose to transfer these three species to the genus *Kryptodasys*. Other morphological traits support the re-classification of these three species according to our proposal. For instance, *M*. *remanei* and *M*. *hexadactylis* both bear paired accessory adhesive tubes arising ventrolaterally, well anterior to the pharyngo-intestinal junction (see Fig. 8 in Boaden^29^ and Fig. 1 in Rao^30^); this is an anatomical characteristic that is also present in all three *Kryptodasys* species described from Sardinia, Brazil and Sweden but not in other species of *Macrodasys*. In addition, *M*. *hexadactylis* possesses short spermatozoa, lacking a typical flagellum (see Fig. 5 in Rao^30^), a condition shared with the three new *Kryptodasys* species but which is in contrast with the filiform spermatozoa of the ‘genuine’ *Macrodasys* taxa. Future research could shed light on the presence of the paired accessory adhesive tubes in *M*. *nobskaensis* and on the morphology of the spermatozoa in *M*. *remanei* and *M*. *nobskaensis*, information that is currently unavailable.

Beside the three species indicated above, another species, currently classified in the genus *Macrodasys*, should be transferred to the genus *Kryptodasys* i.e., *M*. *celticus* Hummon, 2008.

In describing this species, Hummon^31^ remarks: “*Unusual is the female reproductive system of* Macrodasys celticus *n*. *sp*., *eggs developing front to rear*, *combined with a caudal organ that is located just behind the largest egg*, *along with an absence of any structure where the frontal organ would be found*”. Because the combination of such characteristics have no equal among gastrotrichs, if real, they would require the erection of (at least) a new genus to allocate these specimens. However, the situation might be much simpler. In fact, if the two structures thought by the author to be developing oocytes positioned anterior to the largest egg are re-interpreted as the frontal organ, then the organization of the reproductive apparatus of this species is similar to that of species of the genus *Kryptodasys*. The shape and position of the caudal organ along with the arrangement of the TbA in diagonal columns make the resemblance of *M*. *celticus* and *Kryptodasys* species even stronger and strengthen our hypothesis. Again, future investigations should verify in *Kryptodasys celticus* (=*Macrodasys celticus*) the presence of the accessory adhesive tubes and shape of the spermatozoa, information currently lacking.

An emended diagnosis of the genus *Macrodasys* along with a list of the species that fit such a diagnosis, and a taxonomic key to species of the genus *Kryptodasys* are reported in the Supplementary Material, Appendix S1.

### Phylogenetic remarks

In the last two decades, phylogenetic studies using molecular genetic data have proved to be very helpful in clarifying relationships among and within a large number of taxonomic groups. Regarding Gastrotricha, previous phylogenetic studies based on gene sequences (18S rRNA gene alone or in combination with the 28S rRNA and Cox1 genes) have offered support for some of the traditional grouping established on anatomical characteristics (morphological traits) e.g., the families Thaumastodermatidae and Turbanellidae, most genera etc., but have also highlighted unpredicted associations e.g., between species that seem to have very little in common with regard to the general anatomy or between taxa that were affiliated to distinct families^16,32,33^. Some of the new phylogenetic alliances revealed by these early molecular studies have then been confirmed by subsequent investigations, and considered to be very likely by re-assessing in an evolutionary framework the morphological characteristics of the species involved^7,16^. These outcomes have permitted the re-classification of some taxa and consequently reduced some of the major divergences existing between e.g., the traditional classification based on morphological characteristics and the new phylogenetic scenarios put forward by the molecular analyses. In this novel framework, other studies based on molecular data have provided clues about the origin and phylogenetic alliances in cases where the morphological information had proved to be non-resolutive^9,34^ while others have strengthened early hypotheses based on morphological clues^35,36^. These experiences leave little doubts about the high significance of the molecular studies in assessing the evolutionary relationships within Gastrotricha. Results of the present study provide robust evidence (i.e., high congruence of the obtained trees, and high statistical support at the crucial nodes) regarding the status and the phylogenetic position of the new species and genus within Macrodasyidae. In our opinion, this hypothesis should be considered very likely, mostly because in a wider framework, the topology of the current phylogenetic trees confirms the in-group evolutionary scenario of the Macrodasyida illustrated by other recent studies^8,9,16,37^. Moreover, while at a morphological level there are not traits that could suggest different, potential alliances (e.g., armored cuticle, spermiducts confluent in a single male pore etc.), the new species share several similarities with the other members of Macrodasyidae especially with *Macrodasys* (see above). While the position of the new genus within Macrodasyidae appears robust, we point out that in the current analysis (and in others as well), another taxon associated with the Macrodasyidae by traditional taxonomy, *Urodasys*, does not band with the other macrodasyids. This conflicting condition should be explicitly addressed in future research e.g., by improving the taxonomic sampling of *Urodasys* species.

### Conclusive remarks

A growing body of evidence suggests the evolutionary alliances hypothesized by analyses based on molecular markers (e.g., 18S rRNA gene) to be very likely and consequently extremely valuable in the ongoing process of the natural classification of the Gastrotricha.

This is particularly true in the case of robust out-puts e.g., the resulting trees bear high support at nodes and/or the topologies obtained from different analysis are virtually the same. Our current study seems to be strictly in line with all the above. Consequently, the uniqueness of the new species described here, and their place within the Macrodasyidae, as indicated by the phylogenetic trees, appears to be very likely. Furthermore, the phylogenetic position of the new species within the family, most closed to the genus *Thaidasys* (molecular information), and the layout of their reproductive system distinct from that of *Thaidasys* and *Macrodasys* (morphological information) both support the hypothesis that the formal classification of the new species requires the erection of a new genus, which is named named *Kryptodasys*. A review of the relevant literature, found that species belonging to the new established genus *Kryptodasys* were already recorded in the past, but erroneously affiliated to the affine genus *Macrodasys*. A signal that such event may plague other portions of Gastrotricha taxonomic spectrum and, at the same time, an invitation to further challenge the traditional classification with data based on molecular markers, to reach a sole, concordant phylogeny. Finally, data show that *Kryptodasys*, similarly to most other genera of Gastrotricha, is cosmopolitan in distribution (*sensu* Sterrer^38^), suggesting that this very likely applies also to the very few monotypic genera currently known only from restricted areas, often from a single location, a call to extend the research.

## Methods

### Sampling

Specimens belonging to the new genus were found during unrelated investigations in different geographic areas. The very first specimens belonging to the genus were found by MAT in 2001 during investigation at the Elba Island (Italy). Subsequently, animals with similar characteristics were found over the years in: Sardinia (Italy), São Paulo State (Brazil), Alboran Sea (Spain), Florida (USA), Gullmarsfjord (Western Sweden) and Lanzarote (Canary Islands, Spain). In general, specimens were found in low numbers, often less than five specimens; as a consequence, data granting an adequate description are available, to date, only for the animals collected in Sardinia, Brazil and Sweden. These specimens are described herein as three new species. In all cases, animals were found in sublittoral sand collected using 500 ml plastic jars^39^. The Sardinian gastrotrichs were found from the north western coast of the island, in 2005 near Capo Caccia in sediment taken at a depth of 19–30 m, and subsequently in 2010 at Costa Paradiso in sand collected at a depth of 35 m. Sampling was performed by scuba diving at both locations^40^. The Brazilian specimens were found in 2002 in sand from the island of Ilhabela collected at a depth of 2–4 m, while the Swedish worms were found in 2008 at Östersidan at a depth of 0.5–2.5 m. Both the Brazilian and Swedish samples were collected by skin diving^41–43^. Additional details are provided in the type material section of each species.

Use and handling of the animals subject of the present study (Gastrotricha) is not regulated/prohibited; furthermore, collection from the wild does not require special authorization if carried out in public areas (beaches) as it was in the present case.

### Sample processing and morphological analysis

In all cases, the collected samples were transferred to the local field laboratory and processed within one week from collection. Gastrotrichs were extracted daily from aliquots of sediment by the narcotization-decantation technique, using a sea-water isosmotic (6–7%) magnesium chloride solution. The fauna-containing supernatant was then poured directly into a 3–5 cm-diameter Petri dish and scanned for specimens under a Wild M3/M8 dissecting microscope. Individual gastrotrichs were picked out with a micropipette, wholemounted on microscope slides and studied alive under Nomarski optics^44^; specimens were photographed and measured during observation. The following equipment was used: Sardinia, Leitz Dialux 20 microscope equipped with a Nikon 995 digital camera; Brazil, Zeiss Axioscop 2 Plus microscope fitted with a Nikon 995 digital camera; Sweden, Nikon Eclipse 80i microscope equipped with a Nikon Digital Sight DSFi1 digital camera. After identification, some specimens were stored in 95% ethanol for future DNA analysis (see below).

### Species description and illustration

The description of the new species (see also Supplementary Material, Appendix S1) follows the convention of Hummon *et al*.^45^; the position of some morphological characters along the body are given in percentage units (U) of total body length. CorelDRAW® Graphics Suite v. X5 was used to prepare the line art illustrations and to assemble the figures.

### Granulometric analysis and ecology

Study of the substratum was performed following Giere *et al*.^46^. In short, sand from each site was rinsed with tap water and dried in oven at 60 °C for 48 hr. Then, 150 g of dry sediment was analysed by passing through a stack of six sieves with the following phi mesh size: −1, 0, 1, 2, 3, 4, 5. Sieves were shaken for 15 min on a mechanical sieve shaker. The sediment fraction retained on each sieve was weighed to 0.01 g and the resulting data entered into a computer program founded on the calculation of Seward-Thompson & Hails^47^ to obtain the following parameters: mean grain size, sorting coefficient, skewness and kurtosis^48^. Sediment size and sorting classes are based on Wentworth tables^49^. The ecological characteristics of the new species such as frequency and abundance are determined according the rationale originally proposed by Hummon *et al*.^50^ and subsequently implemented in several other studies e.g., Todaro *et al*.^8,14,15^.

### Molecular analysis

The position of the new species along the Macrodasyida phylogenetic tree was inferred from the analysis of the 18S rRNA gene of 45 terminals belonging to the near complete taxonomic spectrum of the order: 45 species of 25 genera in ten families. A basal species of Chaetonotida, *Xenotrichula intermedia* Remane, 1924 (Xenotrichulidae), was selected to be the out-group. Most of the used sequences were obtained from GenBank (Supplementary Table S1). Of the three new species described herein, sequences were obtained only from *Kryptodasys marcocurinii* sp. nov. and *K*. *ulfjondeliusi* sp. nov. In both cases, sequences were derived from single whole specimens; DNA extraction and gene amplification were carried out according to Todaro *et al*.^51^. The PCR products were cleaned with the QIAquick PCR Purification Kit (QIAGEN) and shipped for sequencing to Macrogen, Korea (www.macrogen.co.kr). Sequences were assembled using Staden v1.6.0^52^ and the alignment performed with MUSCLE as implemented in MEGA 6^53^. The final data matrix, which consisted of 48 terminals and 1895 nucleotide characters, was later opportunely formatted and analysed cladistically using three distinct methods: (1) Maximum Parsimony (MP), (2) Maximum Likelihood (ML), and (3) Bayesian inference (BI). MP and ML were conducted using MEGA 6 while BI was run using MrBayes 3.1.2^54^. For ML and BI methods, the General Time Reversible model setting with estimated proportion of invariable sites and gamma distributed rate variation across sites, favoured by both the lnL and the AICc criteria in MEGA 6 and MrModeltest v2.3^55^, was used. In the MP and ML analyses, statistical support for the tree branches was obtained using 1000 bootstrap replicates. For the BI analysis, two independent trails with four chains each were run for six million generations; after a burn-in of 15000 generations, chains were sampled every 100^th^ generation. A consensus tree (50%) was obtained using TreeView^56^.

### Nomenclatural acts

This article meets the requirements of the amended International Code of Zoological Nomenclature^19^as the article itself, and the nomenclature acts it contains have been registered in ZooBank under urn:lsid:zoobank.org:pub:6630A1EA-3197-4780-9DE9-8560C44F6F4A

## Supplementary information

Supplementary material

## Data Availability

All appropriate data created by the authors (i.e., drawings, photographs etc.) are available within the article and/or its supplementary material. Molecular data supporting the findings are openly available from GenBank under the accession numbers reported in Supplementary Table S1.
