# Supplementary material for: Integrated data analysis allows the establishment of a new, cosmopolitan genus of marine Macrodasyida (Gastrotricha)
Source: Sci Rep. 2019 May 29;9:7989. doi: 10.1038/s41598-019-43977-y (PMC6541715; doi:10.1038/s41598-019-43977-y)
Supplement: Supplementary file 1 — Supplementary material [file 41598_2019_43977_MOESM1_ESM.pdf]

SUPPLEMENTARY MATERIAL

Appendix S1

The following nomenclatural acts have been registered in Zoobank, the online registration system for the ICZN. The ZooBank LSIDs (Life Science Identifiers) under: urn:lsid:zoobank.org:pub:6630A1EA-3197-4780-9DE9-8560C44F6F4A

Integrated data analysis allows the establishment of a new, cosmopolitan genus of marine Macrodasyida (Gastrotricha)

M. Antonio Todaro[1], Matteo Dal Zotto[1,2], Tobias Kånneby[3], Rick Hochberg[4]

[1]Dipartimento di Scienze della Vita, Università di Modena and Reggio Emilia, Via Giuseppe Campi, 213/D, I-41125 Modena, Italy;

[2]Consorzio per il Centro Interuniversitario di Biologia Marina e Ecologia Applicata 'G Bacci', v.le Nazario Sauro 4, I-57128 Livorno, Italy;

[3]Lillhagsskolan, Depkens väg 21, 804 26 Gävle, Sweden;

[4]University of Massachusetts Lowell, One University Avenue, Lowell, MA 01854, USA

Taxonomic account

Phylum Gastrotricha Metschnikoff, 1865

Order Macrodasyida Remane, 1925 [Rao & Clausen, 1970]

Family Macrodasyidae Remane, 1924

Genus *Kryptodasys* gen. nov.

urn:lsid:zoobank.org:act:24D72D24-185C-4D2A-8BCF-853C0E5023E

Kryptodasys carlosrochai sp. nov.

urn:lsid:zoobank.org:act:B5F35A4D-1743-435B-B22E-27893FBBD8D7

(Figs S1–S4)

(= nov. gen. nov. spec. Todaro & Rocha, 2004)

Diagnosis. Body elongate, 485–515 μm in total length (TL), and up to 72 μm in width; flattened ventrally and vaulted dorsally, with vacuolated cells along the body margins; epidermal glands generally few, small, scattered along the body. Cuticular covering smooth, devoid of scales and/or spines. Head slightly trapezoidal, bearing noticeable rounded pestle organs in a constriction. Trunk broadest in the mid-gut region, narrowing gently to the anus, then more quickly to the caudum; caudum in the form of a short tail. Sensory hairs arranged singly in lateral and dorsolateral columns along the body, sparsely on the lateral sides of the head. Ventral locomotor ciliature in the form of two bands of sparse and short cilia extending separately from under the head to the posterior trunk region but converging behind the anus into a single band. Anterior adhesive tubes (TbA), three per side, forming diagonal columns inserting directly on the body surface and projecting forward; ventral adhesive tubes (TbV), absent; ventrolateral adhesive tubes (TbVL), up to 10, of which one is in the anterior pharyngeal region (U18) and eight along the intestinal region; third and fourth tubes of the intestinal region separated by a space almost twice as much as the distance between the others. Lateral-, dorsal- and dorsolateral adhesive tubes absent; posterior adhesive tubes (TbP), up to four surrounding the caudum. Paired accessory adhesive tubes, of three tubes per side, arising ventrolaterally from a common base anterior to the pharyngo-intestinal junction at U33. Mouth terminal, rather small (up to 13 μm in diameter), leading to a short buccal cavity (6 μm in length); pharynx up to 175 μm in length and up to 36 μm in width; pharyngeal pores distant from the base with dorsolateral openings at U28. Pharyngo-intestinal junction (PhIJ) at U36. Intestine straight, widest at mid-body (U53); anus ventral at U89. Hermaphroditic; testicles paired, elongate, beginning just anterior to the PhIJ; sperm ducts short, presumably open separately on the ventral

surface; Spermatozoa, squat (4 μm long and 1.5 μm wide), in the form of little nails, apparently lacking a
flagellum. Caudal organ, posterior to the ovary, centered at U77 (Figs S1B, S2D); glando-muscular in nature and approximately bullet-shaped, 54 μm long and 14 μm wide; it bears a canal with a single opening at the posterior end. Frontal organ, sac-like, anterior to the largest oocyte, centered at U51; non muscular in nature and roughly round in shape (20 μm long and 20 μm wide); it shows some internal compartmentalization, with the posterior portion containing a number of spermatozoa while the anterior portion contains secretory material.

Etymology. The species is named after Carlos Rocha, colleague and friend, who organized a series of investigations on marine meiofauna of the State of São Paulo (Brazil), during one of which the species was originally found.

Type specimen. Holotype: the 509 μm long adult specimen shown in Figures S2, S3 no longer extant (International Code of Zoological Nomenclature, Articles 73.1.1 and 73.1.4; see also recommendation 73G–J of Declaration 45 - Addition of Recommendations to Article 73, ICZN 2017), collected on 28/04/2002. *Additional examined material.* Two adult specimens from the same sandy sample; all specimens were observed alive and are no longer extant.

Distribution and ecology. Type locality - Brazil, São Paulo, Praia de Castelhanos, on the east coast of the island of Ilhabela (Lat. 23°51'27.8" S; Long. 45°17'20.5" W); occasional in frequency of occurrence, and scarce in abundance at a depth of 2.0–4.0 m, in fine, poorly sorted sand. values of salinity and temperature of the interstitial water at the time of sampling were 34.8 ‰ and 23° C, respectively. Values of the granulometric parameters are reported in Table S1.

Description. Based mostly on the adult specimen with a total body length of 509 μm (Figs S2, S3). Body elongate and of medium width; flattened ventrally and vaulted dorsally, with vacuolated cells along the lateral and dorsolateral body margins (Figs S1, S2A, S4A); epidermal glands few, small, scattered along the body. Cuticular covering smooth, devoid of scales and/or spines. Head slightly bulbous, bearing rounded pestle organs in a constriction (36 μm) at U04 (Figs S1, S2A, C, D). Body showing two additional constrictions, in the pharyngeal region at U14 (37 μm, and just anterior to PhIJ at U35 (40 μm), then increasing slightly in breadth to mid trunk and there after narrowing gently to the anus, and subsequently more quickly to the caudum; caudum in the form of a short tail (Figs S1, S2A, S4A). Widths of head/mid pharynx/PhIJ/trunk/anus/base of tail, and locations along the length of the body are as follows: 40/43/49/52/25/13 μm at U04/U20/U35/U60/U89/U92, respectively.

Ciliation. Sparse sensorial cilia (8–15 μm in length) insert on the dorsal and ventrolateral margin of the head, in addition about 20–25 sensory hairs (12–20 μm in length) arranged singly in lateral and

dorsolateral columns along the body. Ventral locomotor ciliature forms two longitudinal bands extending separately from under the head to the posterior trunk region but converging behind the anus into a single band; cilia in the bands are rather sparse and short (8–10 µm in length; Fig. 6D).

Adhesive tubes. TbA, three per side (4–6 µm in length), forming diagonal columns, inserting directly on the body surface and projecting forward (Figs S1A, S3D); TbV, absent; TbVL, nine per side (8–13 µm in length), one of which in the anterior pharyngeal region at U18, and eight along the intestinal region from U47 to U89. The third and the fourth tube of the intestinal region are separated by a distance that is almost the double the distance between the others (Fig. S1). TbL, TbDL and TbD absent; TbP, four per side (6–12 µm in length), two of which at the distal end of short tail (Figs S1, S2A). In addition, there are paired accessory adhesive tubes, of three tubes per side, arising, in a row, ventrolaterally from a common base, anterior to the pharyngo-intestinal junction at U33 (Figs S1, S2A, S4). Tubes are of different size, and posterolaterally directed. The longest tube, up to 32 µm in length, emerges in between the other two; the shortest tube, up to 9 µm in length, is the most medial (i.e., closest to the body midline) while the other, up to 14 µm in length, is the most lateral (Figs S1A, S2A, B).

Digestive tract. Mouth is terminal, 13 µm in diameter; buccal cavity rather shallow (6 µm in length) and lined with a thin cuticle (Figs S1B, S2A); pharynx, 173 µm in length, widens toward the rear up to 21 µm; pharyngeal pores open dorsolaterally far off from the base, at U28. Pharyngo-intestinal junction (PhIJ) at U36. Intestine increases slightly in breadth from the PhIJ to mid-body where it reaches 32 µm in width and then gradually narrows toward the posterior body end; anus ventral at U89. In all of the examined specimens, the intestine contained yellowish/orange coloured material, probably biodetritus, but not diatoms frustules (Figs S2A, S3).

Reproductive tract. Hermaphroditic; testicles paired and elongate; they begin just anterior to the PhJI and span posteriorly for about 97 µm, from U35 to U50 (Figs S1B, S2C). Testicles appear to be anatomically and functionally compartmentalized as the anterior portion contains larger and better structured cellular elements (likely spermatozoa), the mid portion shows smaller roundish bodies (likely maturing sperm) while the posterior longer portion holds large cells (primary germinal cells, Figs S1B, S3A). Each testicle apparently extends posteriorly with a short sperm duct, which presumably opens on the ventral surface. Ultrastructural studies are needed to ascertain the exact organization and function of testicles and ducts. Spermatozoa appear stubby (4 µm long and 1.5 µm wide), in the form of little nails, apparently lacking a flagellum (Figs S1B, S3A, C, D). Ovary single, in the second third of the trunk; oocytes maturing in a caudo-cephalic direction with largest oocyte dorsal to the mid intestine centered at U61 (Figs S1B, S3B, C). Caudal organ noticeable, posterior to the ovary, centered at U77 (Figs S1B, S2A, S3B); glando-muscular in nature

and approximately bullet-shaped, 54 µm long and 14 µm wide; it bears a canal with a single opening at its posterior end; the entire organ opens on the ventral surface, anterior to the anus at U82. Frontal organ, sac-like, anterior to the largest oocyte, centered at U51; non muscular in nature and roughly round in shape (20 µm long and 20 µm wide); it shows some internal compartmentalization, with the posterior portion containing a number of immotile spermatozoa while the anterior portion contains secretory material and vacuolated elements (Figs S1B, S3C, D). Neither internal nor external opening were observed.

Variability and remarks. The two additional measured adult specimens ranged from 485 to 515 µm in total length, with a pharynx up to 175 µm in length. Both of them had fully developed male and female gonads, and accessory reproductive organs, similar to the holotype. The adhesive apparatus in general was also similar to that of the holotype, but the 515 µm long adult had an additional TbVL per side in the intestinal region (10 TbVL in total). One of the specimens had the accessory adhesive tubes made up of three tubes each, like the holotype (Fig. S4), the other possessed paired accessory adhesive tubes made up of two tubes instead of three tubes. We think that the normal condition for the species is to have the accessory tubes composed of three tubes and the state exhibited by one of the adults to be an aberration. Factors causing the aberration are unknown. This new species will be differentiated below after the description of the third new species.

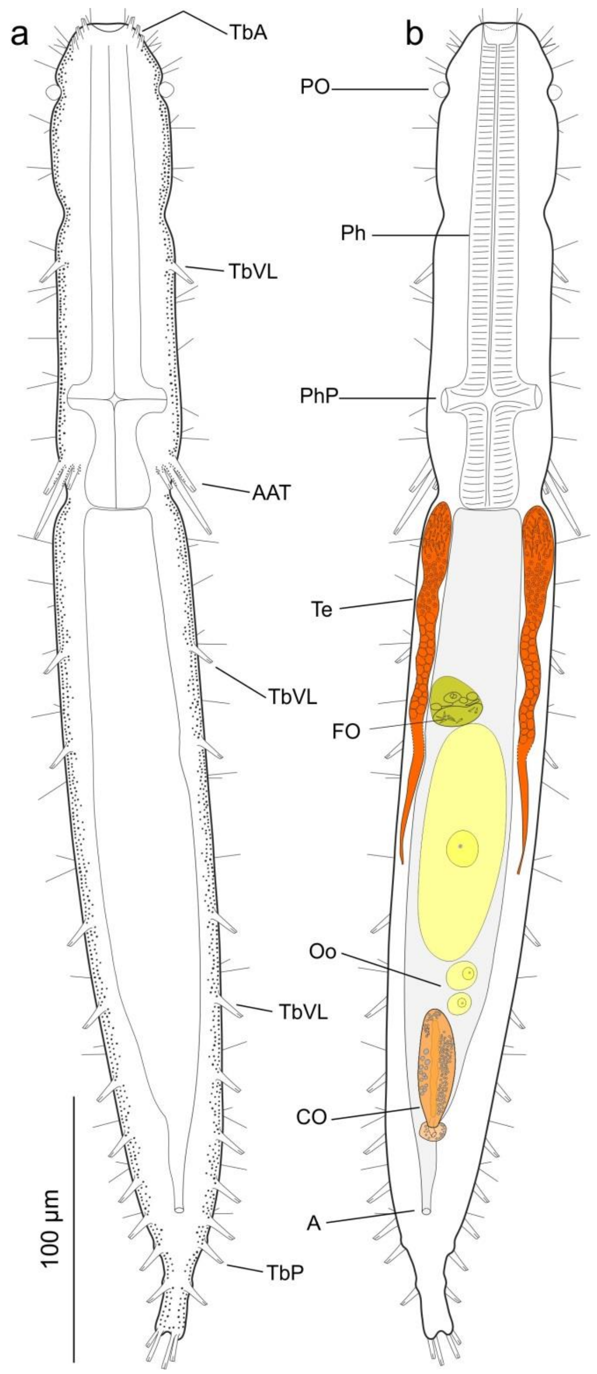

Figure S1. Line art illustrations of *Kryptodasys carlosrochai* sp. nov. (**a**), habitus as seen from the ventral side. (**b**) habitus as seen from the dorsal side, showing the internal anatomy with the male and female reproductive structures. Drawings are made mostly from the holotypic specimen. Abbreviations: A = anus, AAT = accessory adhesive organ, CO = caudal organ, FO = frontal organ, Oo = oocyte, Ph = pharynx, PhIJ = pharyngo-intestinal junction, PhP = pharyngeal pore, PO = pestle organ, TbA = anterior adhesive tube, TbP = posterior adhesive tube, TbVL = ventrolateral adhesive tube, Te = testicle.

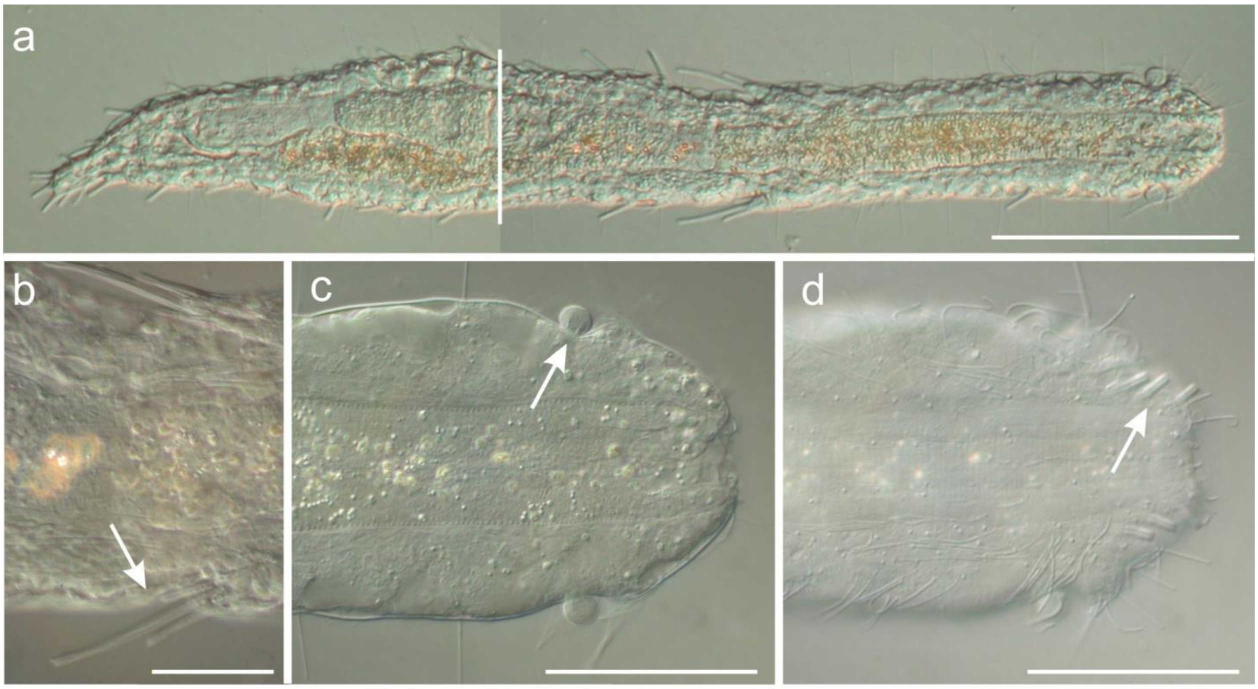

Figure S2. Differential interference contrast photomicrographs, showing the morphology *of Kryptodasys carlosrochai* sp. nov. (**a**) habitus as seen from the ventral side. (**b**) close-up of the posterior pharyngeal region, showing the accessory adhesive organs (arrow). (**c**) close-up of the anterior region, showing the pestle organs (arrow). (**d**) close-up of the anterior region, ventral view, showing the anterior adhesive tubes (arrows). Scale bars (**a)** = 100 µm, (**b)** = 20 µm, (**c**, **d)** = 30 µm.

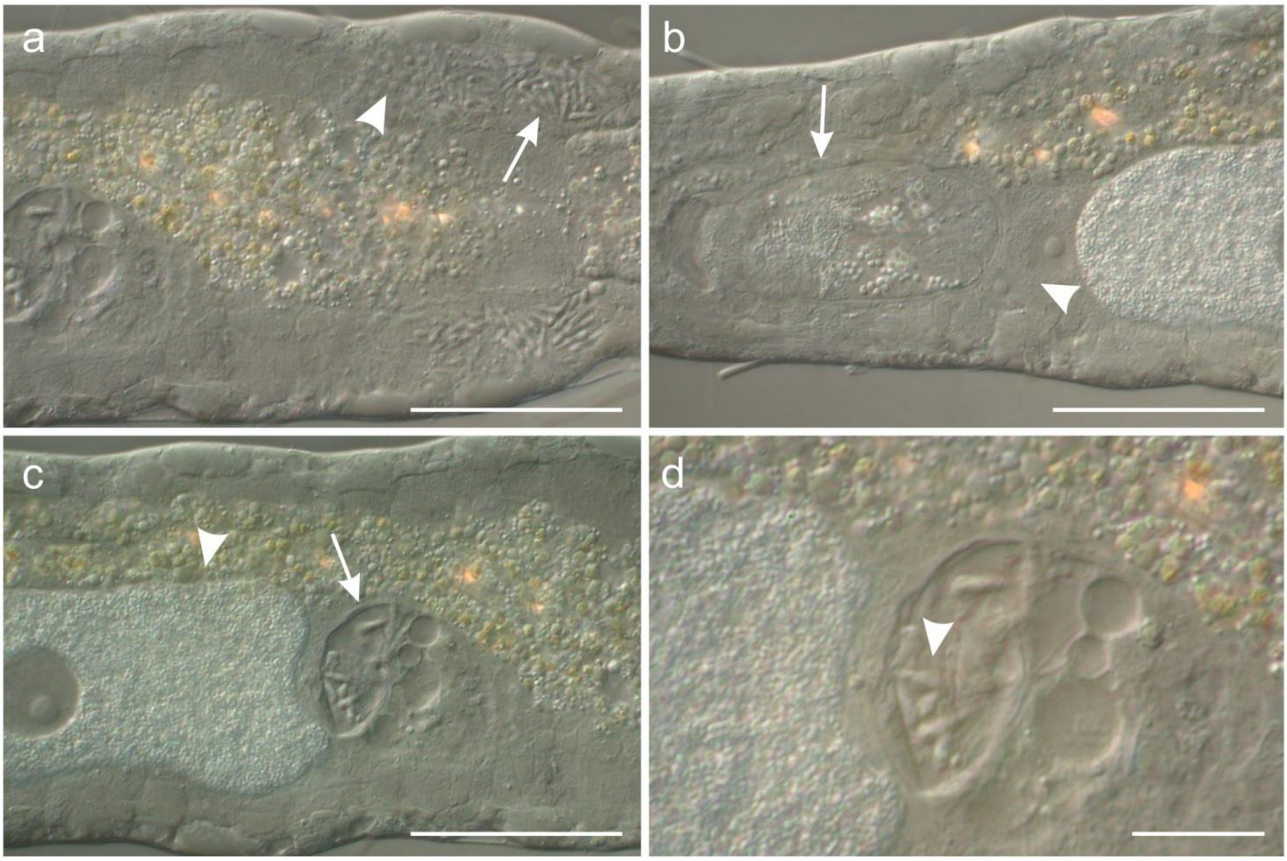

Figure S3. Differential interference contrast photomicrographs, showing gonads and accessory reproductive organs of *Kryptodasys carlosrochai* sp. nov. (**a**), testicles apparently compartmentalized, showing mature spermatozoa (arrow) in the anterior portion followed by small cellular elements, possibly maturing sperm, mixed with granular material (arrowhead). (**b**) posterior trunk region, showing the ovary (arrowhead) followed by the caudal organ (arrow). (**c**) mid trunk region, showing the frontal organ (arrow), anterior to the largest egg (arrowhead). (**d**) close-up of the frontal organ showing some sperm inside. Scale bars (**a**–**c) =** 30 µm, (**d) =** 10 µm.

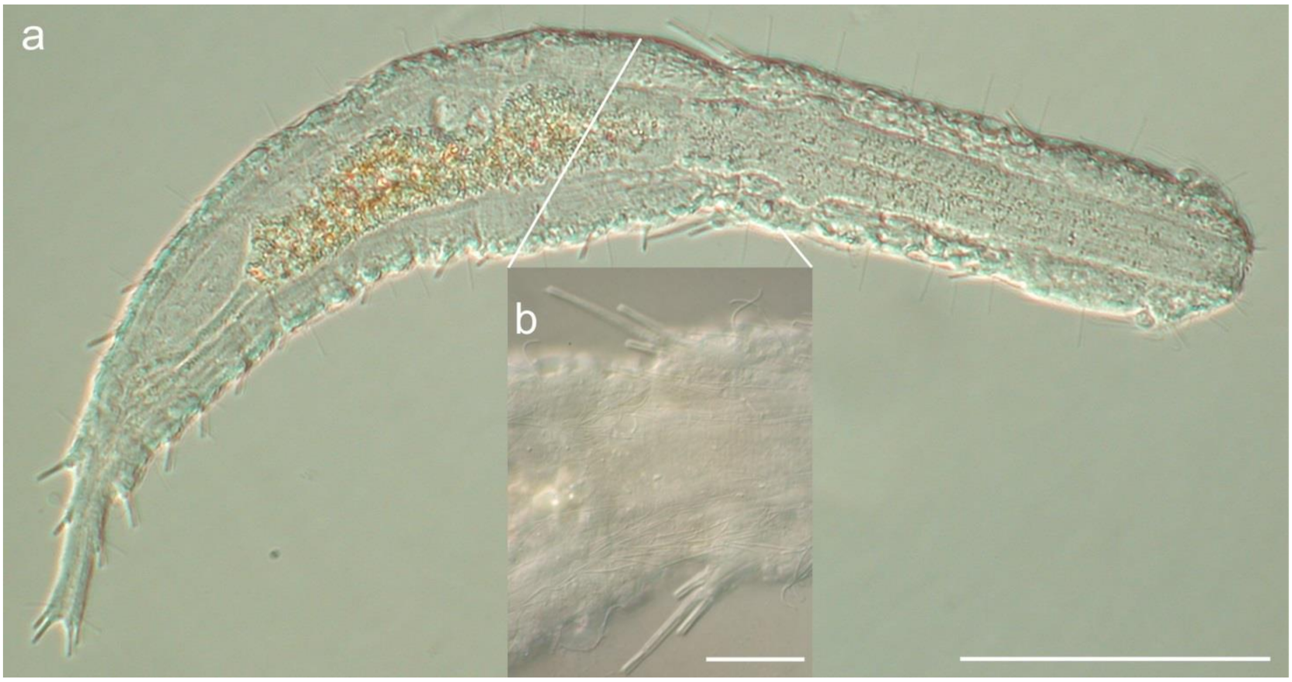

Figure S4. Differential interference contrast photomicrographs of an additional studied adult specimen of *Kryptodasys carlosrochai* sp. nov. (**a**) habitus as seen from the ventral side. (**b**) close-up of the posterior pharyngeal region, showing the accessory adhesive organs. Scale bars (**a**) = 100 µm, (**b**) = 20 µm.

Kryptodasys ulfjondeliusi sp. nov.
urn:lsid:zoobank.org:act:BF9614B6-9D50-4292-B106-A0E1624008D8
(Figs S5–S7)

Diagnosis. Body elongate, 545–595 µm in total length (TL), and up to 71 µm in width; flattened ventrally and vaulted dorsally, with vacuolated cells along the body margins; epidermal glands generally few, small, scattered along the body. Cuticular covering smooth, devoid of scales and/or spines. Head ovoid, bearing rather small, rounded pestle organs in a constriction. Trunk broadest in the mid-gut region, narrowing gently to the anus, then more quickly to the unilobed caudum. Sensory hairs arranged singly in lateral and dorsolateral columns along the body, more densely on dorsal and lateral sides of the head. Ventral locomotor ciliature in the form of two bands of cilia extending separately from under the head to the posterior trunk region but converging behind the anus into a single band. Anterior adhesive tubes (TbA) up to four per side, forming diagonal columns inserting directly on the body surface and projecting forward; ventral adhesive tubes (TbV) absent; ventrolateral adhesive tubes (TbVL), up to 18, three of which in the pharyngeal region and

15 along the intestinal region; TbL, two per side in the pharyngeal region; TbDL absent; TbD, five per side, one in the pharyngeal region and four along the intestinal region. TbP, five per side, two of which at the distal end. In addition, there are paired accessory adhesive tubes, of two tubes per side, arising ventrolaterally from a common base, anterior to the pharyngo-intestinal junction; tubes are rather difficult to see and of different size. Mouth terminal, 19 µm in diameter; buccal cavity mug shaped, 24 µm in length; pharynx up to 233 µm in length and 27 µm in width; pharyngeal pores open dorsolaterally far-off from the base at U32. PhIJ at U43. Intestine straight, wider in the first half; anus ventral at U92. Hermaphroditic; testicles paired, elongate, beginning just anterior to the PhIJ; sperm ducts rather long, that presumably open separately at U67. Spermatozoa, small, apparently lacking a flagellum and shaped like stout spindles. Ovary single, in the posterior third of the body; oocytes maturing in a caudo-cephalic direction, with the largest oocyte centered at about U71. Caudal organ, posterior to the ovary; glando-muscular in nature and approximately bullet-shaped, its opening on the ventral surface, anterior to the anus at U86. Frontal organ, sac-like, anterior to the largest oocyte, centered at U51; non muscular in nature and roughly rounded or wider then longer in shape; usually it contains 1-2 immotile spermatozoa surrounded by secretory material.

Etymology. The species is named after Ulf Jondelius, colleague and friend, who organized a series of investigations on Gastrotricha of Sweden, during one of which the species was originally found.

Type specimen. Holotype: the 593 µm long adult specimen shown in Figure S6 no longer extant (International Code of Zoological Nomenclature, Articles 73.1.1 and 73.1.4; see also recommendation 73G–J of Declaration 45 - Addition of Recommendations to Article 73, ICZN 2017), collected on 24/07/2009. *Additional examined material.* Two adults and one juvenile from the same sandy sample; all specimens were observed alive and are no longer extant. Three additional identified specimens were fixed in 95% ethanol solution, kept in the collection of the first author and later used for DNA analysis (see below).

Distribution and ecology. Type locality - Sweden, Gullmarsfjorden, Östersidan, Klubban beach; on the west coast just outside of the Klubban Biological Station (Uppsala University) (Lat. 58º 15’06’’ N; Long. 11º 27’55’’ E); occasional in frequency of occurrence, and scarce in abundance at a depth of 2.0–4.0 m, in medium, poorly sorted sand with some detritus. Values of salinity and temperature of the interstitial water at the time of sampling were about 26‰ and 20° C, respectively. Values of the granulometric parameters are reported in Table S1.

Description. Based mostly on the adult specimen with a total body length of 593 µm shown in Figure 10. Body elongate and of medium width; flattened ventrally and vaulted dorsally, with

vacuolated cells along the lateral and dorsolateral body margins (Fig. S5, S6A); epidermal glands few, small, scattered along the body. Cuticular covering smooth, devoid of scales and/or spines. Head distinct, ovoid in shape, bearing rounded, rather small, pestle organs in a constriction at U04 (Figs S5, S6A–C, S7A). Body of similar width in the anterior portion, increasing slightly in breadth from just anterior of the PhIJ to mid trunk and then narrowing gently to the unilobed caudum (Figs S5, S6A, C). Widths of head/mid pharynx/PhIJ/trunk/anus, and locations along the length of the body are as follows: 63/59/63/71/40 µm at U07/U25/U43/U57/U92, respectively.

The holotype shows an evident relief in the posterior trunk region at U74; the relief is on the left side when seen from the dorsal side and represent the residual scar of an oviposition, which in these animals occurs by rupture of the body wall.

Ciliation. Sparse sensorial cilia (10–16 µm in length) insert on the dorsal and ventrolateral margin of the head, in addition about 35–50 sensory hairs (18–30 µm in length) arranged singly in lateral and dorsolateral columns along the body. Ventral locomotor ciliature forms two longitudinal bands running separately from under the head to the posterior trunk region but converging behind the anus into a single band; bands are denser and poorly spaced under the head and anterior pharyngeal region (Fig. S6C).

Adhesive tubes. TbA, four per side (5–6 µm in length), forming diagonal columns, inserting directly on the body surface and project forward (Figs S5A, S7C); TbV, absent; TbVL, 18 per side (9–13 µm in length), three of which in the pharyngeal region (two anterior and one posterior to the pharyngeal pore), and 15 along the intestinal region, more or less evenly spaced from the pharyngo-intestinal junction to the anus. TbL, two per side (9 µm in length), in the pharyngeal region anterior to the pharyngeal pores; TbDL absent; TbD, 5 per side (4–6 µm in length), one in the pharyngeal region, anterior to the pharyngeal pores and four along the anterior ¾ of the intestinal region. TbP, five per side (4–10 µm in length), two of which at the distal end (Figs S5, S6A). In addition, there are paired accessory adhesive tubes, of two tubes per side, arising ventrolaterally from a common base, anterior to the pharyngo-intestinal junction at U34. The tubes, which are rather difficult to see, are of different size, and posterolaterally directed. The shortest tube, 10 µm in length, arises anteriorly while the longest is posterior and double of that length, 26 µm (Figs S5A, S6C).

Digestive tract. Mouth is terminal, 19 µm in diameter; buccal cavity is mug shaped, 24 µm in length and lined with a thin cuticle (Figs S5B, S6A, S7A); pharynx, 233 µm in length, widens toward the rear up to 27 µm; pharyngeal pores open dorsolaterally far-off from the base, at U32. Pharyngo-intestinal junction (PhIJ) at U43. Intestine is wider in the first half (35 µm in width) then gradually narrows toward the posterior body end; anus ventral at U92. In all of the examined adult specimens

the intestine contained granular, refringent material, but not diatom frustules (Fig. S6B); in the juvenile specimens, the gut contained a single round microalgal cell (Fig. S7F).

Reproductive tract. Hermaphroditic; testicles paired and elongate; they begin just anterior to the PhIJ and span posteriorly for about 84 μm, from U42 to U56 (Figs S5B, S6A). Each gonad contains mainly larger and better structured cellular elements (likely spermatozoa) in its anterior third; in the following third it shows some putative spermatozoa mixed with granular material, while in the posterior third it appears to be rather empty; it continues in an elongate sperm duct that seems to open on the ventral surface at about U67. Spermatozoa appear stubby (10–11 μm long and 4–6 μm wide), in the form of stout spindles, apparently lacking a flagellum (Figs S5B, S6B, S7B). Ovary single, in the second third of the trunk; oocytes maturing in a caudo-cephalic direction; in the two additional studied adults the largest oocyte was located dorsal to the second half of the intestine, centered at about U71 (Fig. S7A). Caudal organ, posterior to the ovary, centered at U80 (Figs S5B, S6B); glando-muscular in nature and approximately bullet-shaped, 53 μm long and 22 μm wide; it bears a canal with a single opening at its posterior end; the entire organ opens on the ventral surface, anterior to the anus at U86. Frontal organ, sac-like, anterior to the largest oocyte, centered at U51; non muscular in nature and roughly round in shape (20 μm in diameter); it contains 1-2 immotile spermatozoa surrounded by secretory material (Figs S5B, S6B, S7B, C). Neither internal nor external openings were observed.

Variability and remarks. The three additional measured adult specimens ranged from 545 to 598 μm in total length, with the pharynx up to 233 μm in length. All of them had fully developed male and female gonads, and the accessory reproductive organs were, in general, similar to those showed by the holotype (Fig. S7A–D). However, in these animals, the frontal organ was not rounded, like the one shown by the holotype, but appeared to be compressed to some extent along its length (i.e., caudo-cephalic axis), probably due to the pressure exerted on it by the largest, growing oocytes (Fig. S7A, C). The adhesive apparatus in general was also similar to that of the holotype, but the 545 μm long specimen had only three TbA per side and 16 TbVL. Curiously, the same animal showed some asymmetries concerning the adhesive apparatus: the TbP numbered regularly five on one side but four on the other, while the accessory adhesive tubes on the left side included a single tube only instead of two tubes as it is usual for the species.

The measured juvenile specimen was 328 μm in total length (Fig. S7F) with a 143 μm long pharynx (PhIJ at U38). It showed, on each side, two TbA, three TbP and four TbVL, all of which along the intestinal region, starting from the PhIJ. The specimen in addition showed a single TbL at about U21 and the accessory adhesive tubes, of two tubes each, at U42 (Fig. S7F). Unfortunately, no information was acquired with regard to the presence and/or number of dorsal tubes (TbD).

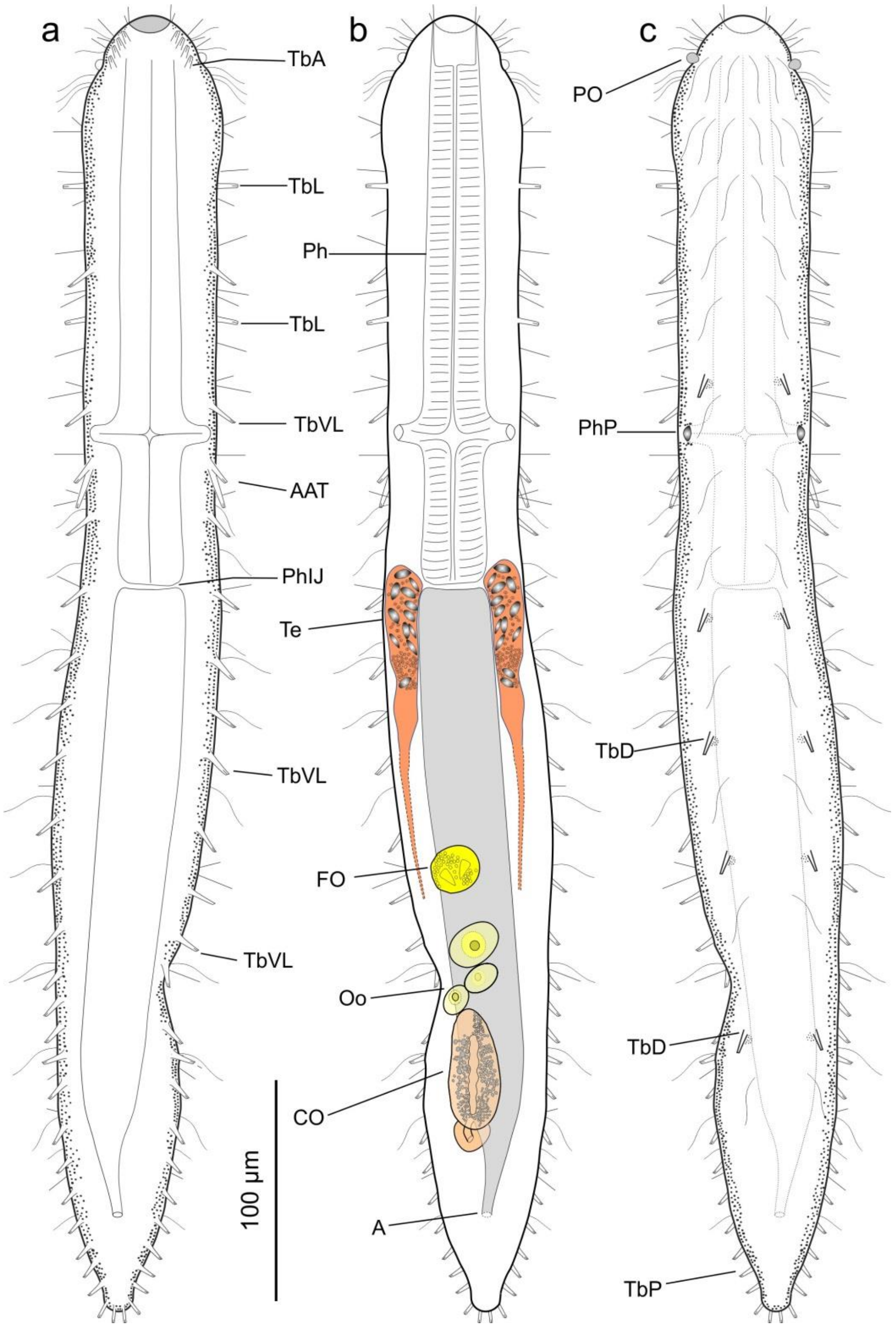

Figure S5. Line art illustrations of *Kryptodasys ulfjondeliusi* sp. nov. (**a**) habitus as seen from the ventral side. (**b**) habitus, showing the internal anatomy with the male and female reproductive structures. (**c**) habitus as seen from the dorsal side. Drawings are made mostly from the holotypic specimen. A = anus, AAT = accessory adhesive tubes, CO = caudal organ, FO = frontal organ, Oo = oocyte, Ph = pharynx, PhIJ = pharyngo-intestinal junction, PhP = pharyngeal pore, PO = pestle organ, TbA = anterior adhesive tube, TbD = dorsal adhesive tube, TbL = lateral adhesive tube, TbP = posterior adhesive tube, TbVL = ventrolateral adhesive tube, Te = testicle.

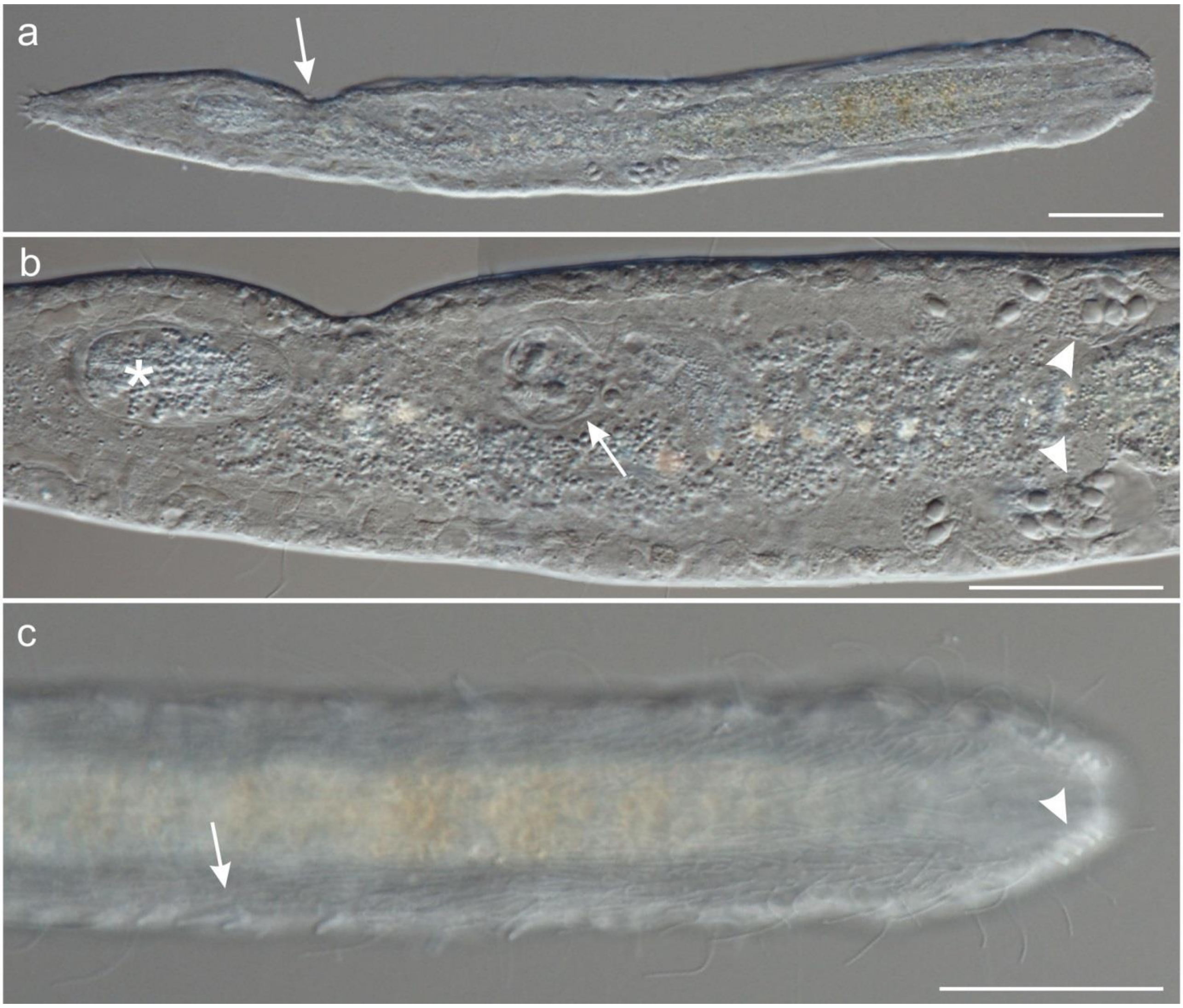

Figure S6. Differential interference contrast photomicrographs, showing the morphology of *Kryptodasys ulfjondeliusi* sp. nov. (**a**) habitus as seen from the dorsal side; the scar seen on the left side (arrow) testifies a recent oviposition which has happened by rupture of the body wall. (**b**) internal anatomy of the trunk region, showing the testicles with stubby spermatozoa (arrowheads), the frontal organ with some sperm inside (arrow) and the caudal organ (asterisk). (**c**) anterior region, ventral view, showing the accessory adhesive tubes (arrow) and the anterior adhesive tubes (arrowhead); Scale bars (**a**) = 100 µm, (**b**, **c**) = 50 µm.

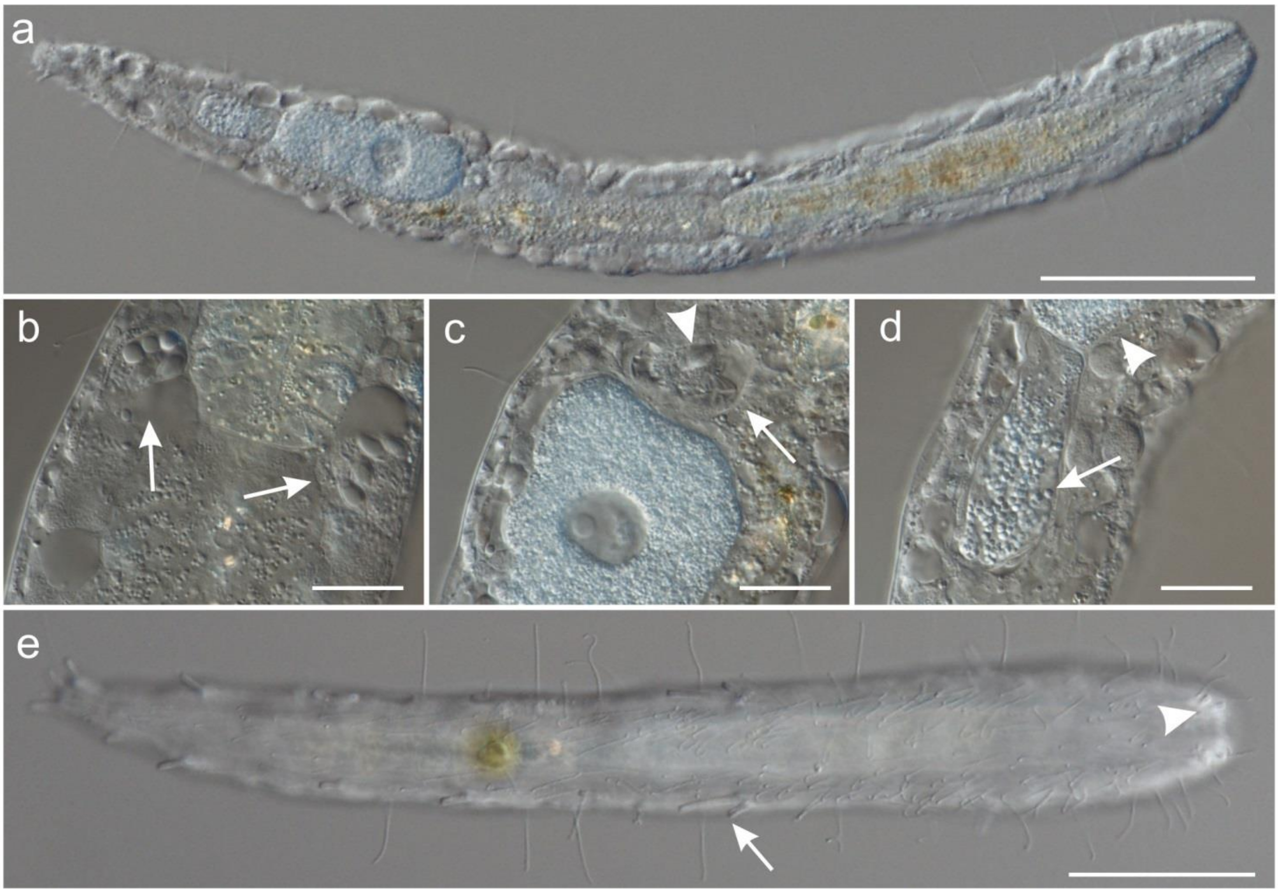

Figure S7. Differential interference contrast photomicrographs of additional studied specimens of *Kryptodasys ulfjondeliusi* sp. nov. (**a**–**d**) adult specimen. (**a**) habitus as seen from the dorsal side. (**b**) testicles with stubby spermatozoa (arrows). (**c**) frontal organ (arrow) anterior the largest egg; some sperm are visible inside the frontal organ (arrowhead). (**d**) caudal organ (arrow) posterior to the ovary (arrowhead). (**e**) subadult specimen, showing the accessory adhesive tubes (arrow) and the anterior adhesive tubes (arrowhead). Scale bars (**a**) = 100 µm, (**b**–**d**) = 20 µm, (**e**) = 50 µm.

Taxonomic key to species of the genus *Kryptodasys*

1 TbDL present **2**
– TbDL absent **4**

2 A single pair of TbD in the pharyngeal region .... ***K. ulfjondeliusi* sp. nov.**
– Three pairs of TbD in the pharyngeal region …………………..………………..…………….. **3**

3 TbD equally spaced along the pharyngeal region .... ***K. celticus* (Hummon, 2008)**
– Two pairs of TbD clustering anterior to the pharyngeal pores and a single pair near the PhIJ ……
…..…………………….…………………………………………..… ***K. nobskaensis* (Hummon, 2008)**

4 Accessory adhesive tubes consist of 3 tubes ……………………..…………...……..…...…. **5**
– Accessory adhesive tubes consist of 2 tubes …………………………….............................. **6**

5 LT up to 515 µm; TbVL up to 10 per side ……….…..………………...… ***K. carlosrochai* sp. nov.**
– LT up to 1000 µm, TbVL up to 30 per side ………..……………… ***K. remanei* (Boaden, 1963)**

6 Five to six pairs of smaller TbVL in the anterior pharyngeal region …… ***K. marcocurinii* sp. nov.**
– TbVL all of similar size …………………………………….……. ***K. hexadactylis* (Rao, 1970)**

***Macrodasys* diagnosis (emended)**

Body elongate, up to 1033 µm in total length (LT), and up to 97 µm in width, flattened ventrally and vaulted dorsally, with vacuolated cells along the body margins; epidermal glands generally few, small, scattered along the body. Cuticular covering smooth, devoid of scales and/or spines. Head slightly ovoid bearing pestle organs, and occasionally ocellar granules. Trunk broadest in the mid-gut region, narrowing gently to the anus, then more quickly to the caudum; caudum unilobed, more often in the form of a short tail. Sensory hairs arranged singly in lateral and dorsolateral columns along the body, sparsely on the lateral sides of the head. Ventral locomotor ciliature covering the entire field or in the form of two bands extending separately from under the head to the posterior trunk region; cilia appear denser in the anterior region. TbA, up to 13 per side, forming an arc or occasionally in two rows, which insert directly on the body surface; TbV, absent; TbL, occasionally present; TbVL, up to 40 per side, some in the pharyngeal region but most along the intestinal region; TbD, rarely present; TbDL, occasionally present in the posterior trunk region; TbP, up to

20, surrounding the caudum. Accessory adhesive tubes, absent. Mouth terminal, of mid-size (up to 27 μm in diameter), leading to a short buccal cavity (8–15 μm in length); pharynx up to 257 μm long and up to 40 μm wide; pharyngeal pores far off from the pharyngeal base, with dorsolateral openings. PhIJ at U30–U45. Intestine increases in width from the PhIJ to mid-body and gradually narrows toward the posterior body end; anus ventral at U85–U94. Hermaphroditic; testicles paired, elongate, usually beginning at the PhIJ; sperm ducts open separately on the ventral surface; spermatozoa, filiform with a cork-screw shaped anterior portion and a long, smooth flagellum. Ovary single, in the second third of the trunk; oocytes maturing in a caudo-cephalic direction with largest oocyte dorsal to the mid intestine. Caudal organ, noticeable, posterior to the ovary; glando-muscular in nature; it bears a canal with a single opening at the posterior end; usually the caudal organ is clearly subdivided in a narrow anterior portion and a wider posterior portion. Frontal organ, just posterior to the largest oocyte; in general compact, occasionally elongate; divided in anterior seminal receptacle and posterior spermatheca. Seminal receptacle with weakly muscolarised wall, showing a small, anterior pore (internal pore), occasionally slightly circularised (nozzle) and a second pore (external pore) that opens on the ventral surface. Type-species *Macrodasys buddenbro*cki Remane, 1924; other species: *M. achradocytalis* Evans, 1994; M. *acrosorus* Hummon & Todaro, 2009; *M. affinis* Remane, 1936; *M. africanus* Remane, 1950; *M. africanus ponticus* Valkanov, 1957; *M. ancocytalis* Evans, 1994; *M. andamanensis* Rao, 1993; *M. balticus* Roszczak, 1939; *M. blysocytalis* Evans, 1994; *M. caudatus* Remane, 1927; *M. cephalatus* Remane, 1927; *M. cunctatus* Wieser, 1957; *M. deltocytalis* Evans, 1994; *M. digronus* Hummon & Todaro, 2009; *M. dolichocytalis* Evans, 1994; *M. fornerise* Todaro & Rocha, 2004; *M. gerlachi* Papi, 1957; *M. gylius* Hummon, 2010; *M. imbricatus* Hummon, 2011; *M . indicus* Kutty & Nair, 1969; *M. lakshadweepense* Hummon, 2008; *M. macrurus* Hummon, 2011; *M. meristocytalis* Evans, 1994; *M. neapolitanus* Papi, 1957; *M. nigrocellus* Hummon, 2011; *M. ommatus* Todaro & Leasi, 2013; *M. pacificus* Schmidt, 1974; *M. plurosorus* Hummon, 2008; *M. scleracrus* Hummon, 2011; *M.*

stenocytalis Evans, 1994; *M. syringodes* Hummon, 2010; *M. thuscus* Luporini, Magagnini & Tongiorgi, 1973; *M. waltairensis* Rao & Ganapati, 1968.

SUPPLEMENTARY MATERIAL

Tables

Table S1. Gastrotrich taxa involved in the molecular analyses. GenBank accession number, geographic origin and reference.

Taxon	Accession	Origin	Reference
**Cephalodasyidae**			
Cephalodasys sp.1	AY963691	White Sea, Russia	Petrov et al., 2007
Dolichodasys sp. 1	AM231778	San Isidoro, Italy	Todaro et al., 2006
Mesodasys laticaudatus	JF357657	Albinia, Italy	Todaro et al., 2011
Mesodasys littoralis	JF357658	Bou Ficha, Tunisia	Todaro et al., 2011
Paradasys sp. 1	AM231781	Ionian sea, Italy	Todaro et al., 2006
Pleurodasys helgolandicus	JN203486	Ibiza, Spain	Todaro et al., 2012
**Dactylopodolidae**			
Dactylopodola cf. *baltica*	JF357650	Ras Alard, Kuwait	Todaro et al., 2011
Dactylopodola mesotyphle	JF357651	Punta Ala, Italy	Todaro et al., 2011
Dactylopodola typhle	JF357652	Bou Ficha, Tunisia	Todaro et al., 2011
Dactylopodola typhle	JF357653	Torre Civette, Italy	Todaro et al., 2011
**Hummondasyidae**			
Hummondasys jamaicensis	KM083602	Negril, Jamaica	Todaro et al., 2014
**Lepidodasyidae**			
Lepidodasys unicarenatus	JF357665	Pianosa, Italy	Todaro et al., 2011
**Macrodasyidae**			
Kryptodasys macrocurinii	MK880150	Sardinia, Italy	Present study
Kryptodasys ulfjondeliusi	MK880151	Gullmarsfiord, Sweden	Present study
Macrodasys sp. 1	JF357654	Torre Civette, Italy	Todaro et al., 2011
Macrodasys sp. 2	JF357670	Bohuslän, Sweden	Todaro et al., 2011
Thaidasys tongiorgii	KR072683	Phuket Island, Thailand	Todaro et al., 2015
Urodasys sp. 1	DQ079912	Florida, USA	Sørensen et al., 2006
Urodasys sp. 2	AY218102	NA	Giribet et al., 2004
**Planodasyidae**			
Crasiella sp.1	JN203488	Ilhabela, Brazil	Todaro et al., 2012
Megadasys sp. 1	JF357656	Porto Cesareo, Italy	Todaro et al., 2011
Megadasys sp. 2	JF357655	Grotta del Ciolo, Italy	Todaro et al., 2011
**Redudasyidae**			
Anandrodasys agadasys	JN203487	St. John Island, USA	Todaro et al., 2012
Redudasys fornerise	JN203489	Represa do Broa, Brazil	Todaro et al., 2012
**Thumastodermatidae**			
Acanthodasys sp. 1	JF357638	Capraia, Italy	Todaro et al., 2011
Acanthodasys aculeatus	JF357639	Capraia, Italy	Todaro et al., 2011
Diplodasys ankeli	JF357624	Meloria, Italy	Todaro et al., 2011
Diplodasys meloriae	JF357640	Meloria, Italy	Todaro et al., 2011
Oregodasys ocellatus	JF357642	Meloria, Italy	Todaro et al., 2011

Oregodasys ruber	JF357625	Meloria, Italy	Todaro et al., 2011
Oregodasys tentaculatus	JF357626	Meloria, Italy	Todaro et al., 2011
Pseudostomella etrusca	JF357633	Albinia, Italy	Todaro et al., 2011
Ptychostomella lamelliphora (=sp1)	JF357643	Ilhabela, Brazil	Todaro et al., 2011
Ptychostomella tyrrhenica	JF357634	Albinia, Italy	Todaro et al., 2011
Tetranchyroderma esarabdophorum	JF357627	Mahdia, Tunisia	Todaro et al., 2011
Tetranchyroderma hirtum	JF357628	Capraia, Italy	Todaro et al., 2011
Tetranchyroderma papii	JF357637	Sardegna, Italy	Todaro et al., 2011
Tetranchyroderma thysanophorum	JF357630	Albinia, Italy	Todaro et al., 2011
Thaumastoderma moebjergi	JF357671	Bohuslän, Sweden	Todaro et al., 2011
Thaumastoderma ramuliferum	JF357631	Meloria, Italy	Todaro et al., 2011
**Turbanellidae**			
Paraturbanella dohrni	JF357659	Punta Ala, Italy	Todaro et al., 2011
Paraturbanella pallida	JF357660	Capraia, Italy	Todaro et al., 2011
Paraturbanella teissieri	JF357661	Punta Ala, Italy	Todaro et al., 2011
Turbanella bocqueti	JF357662	Tramore, Ireland	Todaro et al., 2011
Turbanella cornuta	JF357663	Chioggia, Italy	Todaro et al., 2011
Turbanella lutheri	JF357669	Torö, Sweden	Todaro et al., 2011
**Xenodasyidae**			
Xenodasys riedli	JN203490	St. John Island, USA	Todaro et al., 2012
**Xenotrichulidae***			
Xenotrichula intermedia	JF357664	Mahdia, Tunisia	Todaro et al., 2011

* Order Chaetonotida; NA, Data not available.

Table S2. Granulometric parameters of the sediment at the investigated locations where the three new *Kryptodasys* species were found.

Location	Mean grain size (phi)	Sorting	Skewness	Kurtosis
**Sardinia, Italy**				
Grotta di Nereo	1.28	0.88	1.25	2.76
Grotta il Porticato	0.51	0.84	0.80	2.93
Costa Paradiso	-0.02	0.48	1.22	7.19
**Ilhabela, Brazil**				
Praia de Castelhanos	2.02	1.10	-1.04	2.70
**Gullmarsfjorden, Sweden**				
Klubban beach	1.84	1.22	-0.52	3.52

Table S3. Number of specimens found and studied/measured, along with the main traits of the adults of the three new *Kryptodasys* species described in the present article; measurements in µm.

	K. marcocurinii	*K. carlosrochai*	*K. ulfjondeliusi*
Specimens found	8	3	7
Specimens studied/measured	4 adults, 1 subadult, 1 juvenile	3 adults	3 adults, 1 juvenile
**Traits of the adults**			
Total length	711-734	485-515	545-598
Pharynx length	227	173-175	230-233
PhIJ at U	33	36	43
TbA per side	5-6	3	3-4
TbVL per side	24-25	Up to 10	16
TbVL in the pharyngeal region	5-6	1	3
TbL per side	1	absent	2
TbD per side	absent	absent	5
TbP total	8-10	4	9-10
N. tubes in the accessory adhesive organs	2	3	2

References

Boaden, P. J. S. The interstitial fauna of some north Wales beaches. *J. Mar. Biol. Ass. U. K.* **43**, 79–96 (1963).

Giribet, G., Sørensen, M. V., Funch, P., Kristensen, R. M. & Sterrer, W. Investigations into the phylogenetic position of Micrognathozoa using four molecular loci. *Cladistics* **20**, 1–13 (2004).

Hummon, W. D. Gastrotricha of the North Atlantic Ocean: 1. Twenty four new and two redescribed species of Macrodasyida. *Meiofauna Mar.* **16**, 117–174 (2008).

ICZN. International code of zoological nomenclature. Fourth Edition. The International Trust for Zoological Nomenclature, London (1999).

ICZN. Declaration 45 - Addition of Recommendations to Article 73 and of the term “specimen, preserved” to the Glossary. *Bull. Zool. Nomencl.* **73**, 2–4 (2017).

Petrov, N. B. et al. Molecular phylogeny of Gastrotricha on the basis of a comparison of the 18S rRNA genes: rejection of the hypothesis of a relationship between Gastrotricha and Nematoda. *Mol. Biol.* **41**, 445–452 (2007).

Rao, G. C. Three new interstitial gastrotrichs from Andhra coast, India. *Cah. Biol. Mar.* **11**, 109–120 (1970).

Sørensen, M. V., Sterrer, W. & Giribet, G. Gnathostomulid phylogeny inferred from a combined approach of four molecular loci and morphology. *Cladistics* **22**, 32–58 (2006).

Todaro, M. A., Dal Zotto, M., Jondelius, U., Hochberg, R., Hummon, W. D., Kånneby, T. & Rocha, C. E. F. Gastrotricha: A marine sister for a freshwater puzzle. *Plos one* **7**, e31740 (2012).

Todaro, M. A., Dal Zotto, M. & Leasi, F. An integrated morphological and molecular approach to the description and systematisation of a novel genus and species of Macrodasyida (Gastrotricha). *Plos one* **10**, e0130278 (2015).

Todaro, M. A., Kånneby, T., Dal Zotto, M. & Jondelius, U. Phylogeny of Thaumastodermatidae (Gastrotricha: Macrodasyida) inferred from nuclear and mitochondrial sequence data. *Plos one* **6**, e17892 (2011).

Todaro, M. A., Leasi, F. & Hochberg, R. A new species, genus and family of marine Gastrotricha from Jamaica, with a phylogenetic analysis of Macrodasyida based on molecular data. *Syst. Biodivers.* **12**, 473–488 (2014).

Todaro, M. A. & Rocha, C. E. F. Diversity and distribution of marine Gastrotricha along the northern beaches of the state of São Paulo (Brazil), with description of a new species of *Macrodasys* (Macrodasyida, Macrodasyidae). *J. Nat. Hist.* **38**, 1605–1634 (2004).

Todaro, M. A., Telford, M. J., Lockyer, A. E. & Littlewood, D. T. J. Interrelationships of the Gastrotricha and their place among the Metazoa inferred from 18S rRNA genes. *Zool. Scr.* **35**, 251–259 (2006).
